# Accuracy of Machine Learning Models for Early Prediction of Major Cardiovascular Events Post Myocardial Infarction: A Systematic Review and Meta-Analysis

**DOI:** 10.31083/RCM37224

**Published:** 2025-06-17

**Authors:** Yi Xiang, Dong Liu, Leilei Guo, Yuhua Zheng, Xiaoman Xiong, Tao Xu

**Affiliations:** ^1^School of Postgraduate Students, Guizhou University of Traditional Chinese Medicine, 550000 Guiyang, Guizhou, China; ^2^School of Medicine, Guizhou University of Traditional Chinese Medicine, 550000 Guiyang, Guizhou, China; ^3^Cardiovascular Medicine, The Second Affiliated Hospital of Guizhou University of Traditional Chinese Medicine, 550000 Guiyang, Guizhou, China

**Keywords:** myocardial infarction, machine learning, MACEs, PCI

## Abstract

**Background:**

Major adverse cardiovascular events (MACEs) significantly affect the prognosis of patients with myocardial infarction (MI). With the widespread application of machine learning (ML), researchers have attempted to develop models for predicting MACEs following MI. However, there remains a lack of evidence-based proof to validate their value. Thus, we conducted this study to review the ML models’ performance in predicting MACEs following MI, contributing to the evidence base for the application of clinical prediction tools.

**Methods:**

A systematic literature search spanned four major databases (Cochrane, Embase, PubMed, Web of Science) with entries through to June 19, 2024. With the Prediction Model Risk of Bias Assessment Tool (PROBAST), the risk of bias in the included models was appraised. Subgroup analyses based on whether patients had percutaneous coronary intervention (PCI) were carried out for the analysis.

**Results:**

Twenty-eight studies were included for analysis, covering 59,392 patients with MI. The pooled C-index for ML models in the validation sets was 0.77 (95% CI 0.74–0.81) in predicting MACEs post MI, with a sensitivity (SEN) and specificity (SPE) of 0.78 (95% CI 0.73–0.82) and 0.85 (95% CI 0.81–0.89), respectively; the pooled C-index was 0.73 (95% CI 0.66–0.79) in the validation sets, with an SEN of 0.75 (95% CI 0.67–0.81) and an SPE of 0.84 (95% CI 0.75–0.90) in patients who underwent PCI. Logistic regression was the predominant model in the studies and demonstrated relatively high accuracy.

**Conclusions:**

ML models based on clinical characteristics following MI, influence the accuracy of prediction. Therefore, future studies can include larger sample sizes and develop simplified tools for predicting MACEs.

**The PROSPERO registration:**

CRD42024564550, https://www.crd.york.ac.uk/PROSPERO/view/CRD42024564550.

## 1. Introduction

Myocardial infarction (MI) is the most severe manifestation among coronary atherosclerotic heart disease. It is mainly caused by the rupture of atherosclerotic plaques, leading to thrombosis and further causing myocardial ischemic necrosis [1]. Although the incidence rate has decreased in developed countries, there are still more than 7 million cases globally every year [2, 3]. The incidence rate continues to rise especially in densely populated countries, which becomes a major public health problem [4, 5]. Currently, although percutaneous coronary intervention (PCI) and other reperfusion therapies have significantly reduced the mortality rate [6, 7, 8], there is still an increased risk of major adverse cardiovascular events (MACEs) after the operation, including reinfarction, stroke, cardiogenic death and others [9]. Therefore, early monitoring of the risk of MACEs is regarded has havingimportant clinical significance.

Currently, widely used clinical risk scoring tools, such as the Global Registry of Acute Coronary Events (GRACE), the Korea Acute Myocardial Infarction Registry (KAMIR), and the Thrombolysis in Myocardial Infarction (TIMI), can guide identifying high-risk patients in clinical practice. Among these, GRACE and TIMI scores are widely recommended as risk assessment tools for populations with acute coronary syndrome (ACS) [10, 11]. Nevertheless, conventional risk assessment techniques possess shortcomings, and early identification methods for patients prone to MACEs are inadequate. Although some studies have explored the predictive value of GRACE and TIMI scores for MACEs, there is insufficient systematic evidence of their predictive performance [12, 13]. Some researchers have focused on the potential value of machine learning (ML) regarding MI in light of the increasing usage of ML techniques in clinical practice, particularly in the context of cardiovascular disorders. For instance, a study by Fatemeh Zabihollahy* et al*. [14] discussed how the combination of ML and cardiac magnetic resonance imaging could optimize diagnostic accuracy and prognostic stratification of patients with MI. Another review by Jun Hua Chong *et al*. [15] highlighted the role of ML in improving the efficiency of clinicians in diagnosing and treating MI, as well as in enhancing the quality of care. Thus, it appears that ML holds promising predictive value in the diagnosis and treatment of MI.

This study aimed to systematically analyze the performance of different ML models in predicting MACEs after MI, with a focus on analyzing the differences among different algorithms in terms of discriminative ability (c-index), accuracy of risk stratification (sensitivity/specificity) and clinical applicability, to further provide an evidence-based basis for optimizing existing risk assessment tools.

## 2. Methods

### 2.1 Study Registration

As per the guidelines of the Preferred Reporting Items for Systematic Reviews and Meta-Analyses (PRISMA 2020), this study was completed and prospectively registered with PROSPERO (ID: CRD42024564550).

### 2.2 Eligibility Criteria

#### 2.2.1 Inclusion Criteria

(1) Studies with a population consisting of patients with MI;

(2) Case-control, cohort, cross-sectional studies;

(3) Studies that established comprehensive predictive models for MACEs in MI patients;

(4) Studies in English.

#### 2.2.2 Exclusion Criteria

(1) Studies that did not distinguish between MI and other heart diseases;

(2) Meta-analyses, reviews, guidelines, expert opinions, conference abstracts without peer review;

(3) Studies that only analyzed differential factors without building a complete ML model;

(4) Studies lacking key outcome measures regarding the accuracy of ML models (accuracy, c-index, confusion matrix, F1 score, precision, receiver operating characteristic (ROC), sensitivity, specificity);

(5) Studies with insufficient sample sizes (<20 cases).

### 2.3 Data Sources and Search Strategy 

Four databases (Cochrane, Embase, PubMed, and Web of Science) were systematically searched up until June 19, 2024, with subject headings + free-text terms. No restrictions were applied regarding geography or age. The detailed search strategy is provided in **Supplementary Table 1**.

### 2.4 Study Selection and Extraction of Data

All retrieved studies were imported into the management software, EndNote, and screened via titles or abstracts to exclude duplicates. Original studies initially aligned with the research were selected, and the complete texts were retrieved for review, to include final original studies that met the criteria. A pre-specified structured data extraction form was formulated prior to data extraction. The extracted information included: title, digital object identifier (DOI), first author, year of publication, country of the authors, study type, patient source, treatment background, definition of MACEs, follow-up duration, number of MACEs, total number of cases, number of MACEs in the training set, total number of cases in the training set, method of validation set generation, overfitting prevention methods, number of MACEs in the validation set, number of total cases in the validation set, methods for handling missing data, methods for feature selection, variable screening, type of model used, and modeling variables.

Two researchers worked independently to screen the literature and gather the data, and discrepancies were resolved through cross-checking. If disagreements arose, a third researcher was consulted for adjudication.

### 2.5 Risk of Bias in Studies

We utilized the PROBAST to examine potential bias in the original studies. The four key categories covered by this instrument are (1) participants, (2) predictors, (3) outcomes, and (4) analysis. The evaluated domains reflect the overall risk of bias and applicability. Each of these four domains includes multiple specific questions, with three possible responses for each question (yes/probably yes vs. no/probably no vs. no information). The overall risk of bias was classified according to pre-specified criteria: studies were deemed ‘low risk’ when all domains achieved low-risk ratings, whereas the presence of high risk in any single domain resulted in an overall ‘high-risk’ classification.

A cross-check was carried out after two researchers used PROBAST to independently evaluate the risk of bias. In the event of a disagreement, adjudication was made by a third researcher.

### 2.6 Synthesis Methods

#### 2.6.1 Systematic Review Process

This systematic review was carried out in strict accordance with the PRISMA 2020 guideline specifications. The screening of titles/abstracts and full texts was independently completed by two researchers (using the pre-determined inclusion criteria in Section 2.2), any discrepancies were resolved through arbitration by a third-party reviewer. Data were extracted by a standardized form, including: study design, population characteristics, types of ML model, definition of outcome indicators and performance parameters (C-index, sensitivity, specificity). The assessment of the risk of bias was conducted by the PROBAST tool (as shown in Section 2.5).

#### 2.6.2 Meta-Analysis Process

The Meta-analysis was conducted by the c-index and the Diagnostic 2 × 2 Table. During the Meta-analysis of the c-index, the consistency among various models were assessed by I^2^. When the I^2^ value was greater than 50%, a random effects model was adopted, while when the I^2^ value was less than 50%, a fixed effects model was adopted. For studies that did not report the confidence intervals, the method proposed by Debray* et al*. [16] was applied to estimate their standard errors. The publication bias of the c-index among various models were assessed by the Funnel plots, and the consistency among the models were evaluated by the Egger’s test. The sensitivity and specificity indicators were synthesized through a bivariate mixed-effects model. When the fourfold table data could not be obtained, the calculation was carried out based on combining sensitivity, specificity, precision and others with the number of cases. All statistical analyses were completed by Stata 15.0 software (StataCorp, College Station, TX, USA).

## 3. Results

### 3.1 Study Selection

**Fig. 1.**
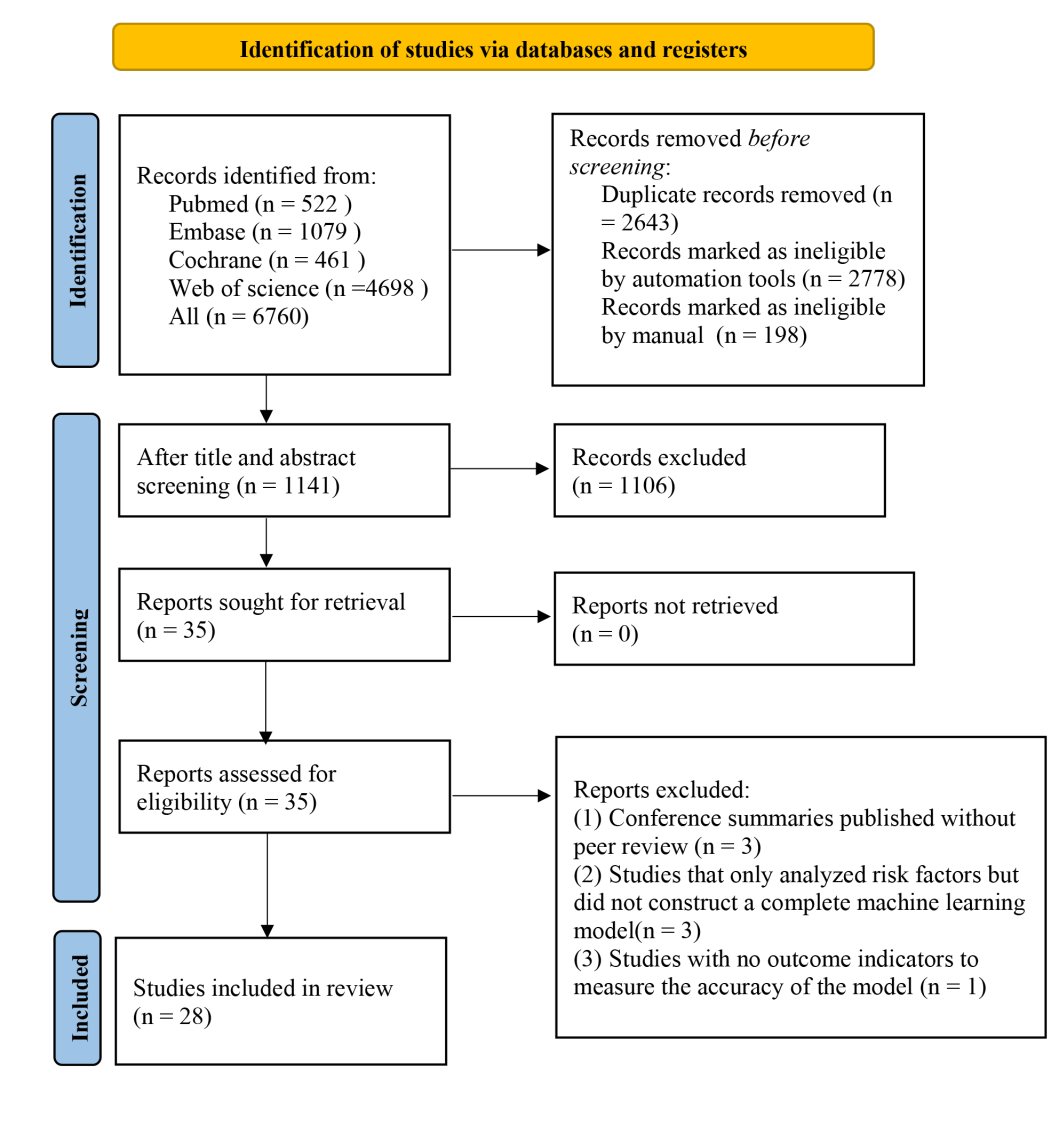
**Literature process by manual screening**.

In total, 6760 studies were retrieved from the database, 5619 of them were duplicates. After duplication removal, 1106 articles were removed through title/abstract screening. For the remaining 35 articles, full texts were downloaded, with 3 articles unable to be retrieved in full. After reviewing the full texts, 4 studies were excluded due to duplicate publications of the same randomized controlled trial with different outcomes or populations, or for lacking the outcome measure(s) of interest. Ultimately, 28 studies [17, 18, 19, 20, 21, 22, 23, 24, 25, 26, 27, 28, 29, 30, 31, 32, 33, 34, 35, 36, 37, 38, 39, 40, 41, 42, 43, 44] were included (Fig. 1).

### 3.2 Study Characteristics

All of the 28 included studies were published between 2019 and 2024. All of them were cohort studies. 9 studies [20, 25, 27, 29, 31, 35, 36, 43, 44] were multicenter, one study [21] was based on a registry database, and the remaining 18 studies [17, 18, 19, 22, 23, 24, 26, 28, 30, 32, 33, 34, 37, 38, 39, 40, 41, 42] were single-center studies. In 19 studies [17, 18, 19, 20, 21, 22, 23, 24, 27, 28, 29, 30, 32, 33, 38, 39, 40, 43, 44], patients were clearly documented as having undergone PCI. In 1 study [35], patients underwent either PCI or balloon angioplasty. The remaining 8 studies [17, 21, 22, 28, 29, 34, 38, 40] did not specify the treatment background of the study population. Follow-up varied from 90 days to 3 years. In total, 59,392 patients were included. Validation methods were described in 21 studies [17, 18, 19, 20, 21, 22, 24, 25, 28, 29, 31, 32, 33, 34, 35, 36, 38, 40, 42, 43, 44]. External validation was used in 6 studies [20, 25, 35, 36, 43, 45], random sampling was applied in 8 studies [17, 21, 22, 28, 29, 34, 38, 40], and k-fold cross-validation or Bootstrap validation was applied in 7 studies [18, 19, 24, 31, 32, 33, 42]. Missing data handling methods were reported in 11 studies [18, 19, 21, 25, 28, 31, 34, 36, 42, 43, 44]. Among them, 7 studies [19, 21, 28, 31, 42, 43, 44] used deletion methods, I study [25] adopted mean imputation, and 3 studies [18, 34, 36] applied multiple imputation. As for variable selection methods, 10 studies [17, 19, 20, 24, 28, 29, 33, 40, 42, 43] applied LASSO regression, while the remaining studies used univariate or multivariate logistic regression. A total of 11 different models were established in the included studies. Details are presented in **Supplementary Table 2**.

### 3.3 Risk of Bias in Studies

**Fig. 2.**
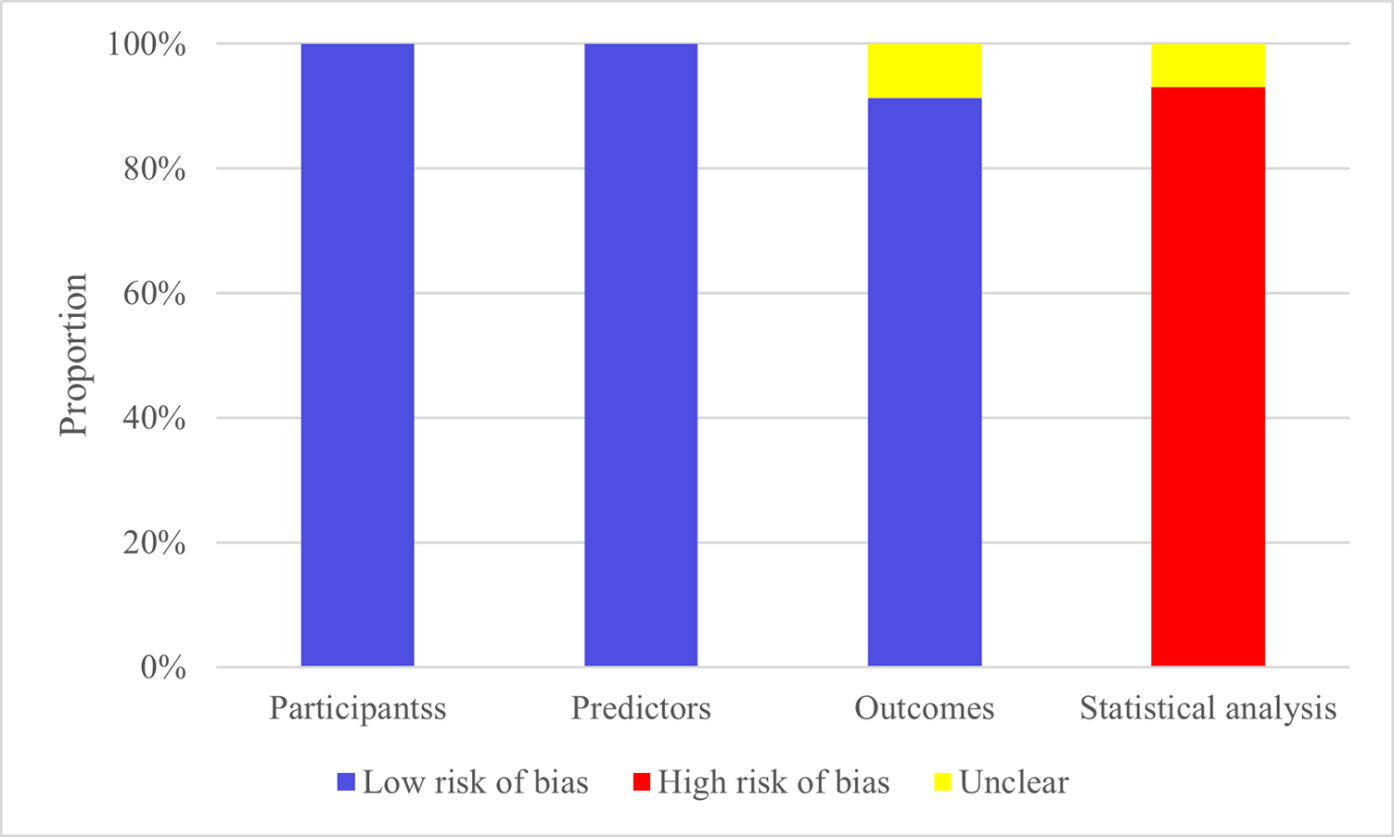
**Risk of bias assessment results of the models**.

Studies included were all cohort studies or from registry databases, resulting in a low risk of bias regarding the study population. All studies assessed predictors without knowing the outcomes and had less than 40% missing data, resulting in a low risk of bias. Regarding outcomes, some models did not report whether predictor information was unclear at the time of outcome determination, so these were marked as having no information, while the others were deemed low risk. Regarding statistical analysis, 5 models failed to meet the requirement of events per variable (EPV) ≥10 or lacked an independent validation set, thus representing a high risk of bias. Additionally, 7 models that handled missing data using deletion methods were also considered high risk. Six models that selected predictors based on univariate analysis were also rated as high risk. Detailed assessment results are provided in Fig. 2.

### 3.4 Meta-Analysis

#### 3.4.1 All Patients

3.4.1.1 Synthesized Results

**Fig. 3.**
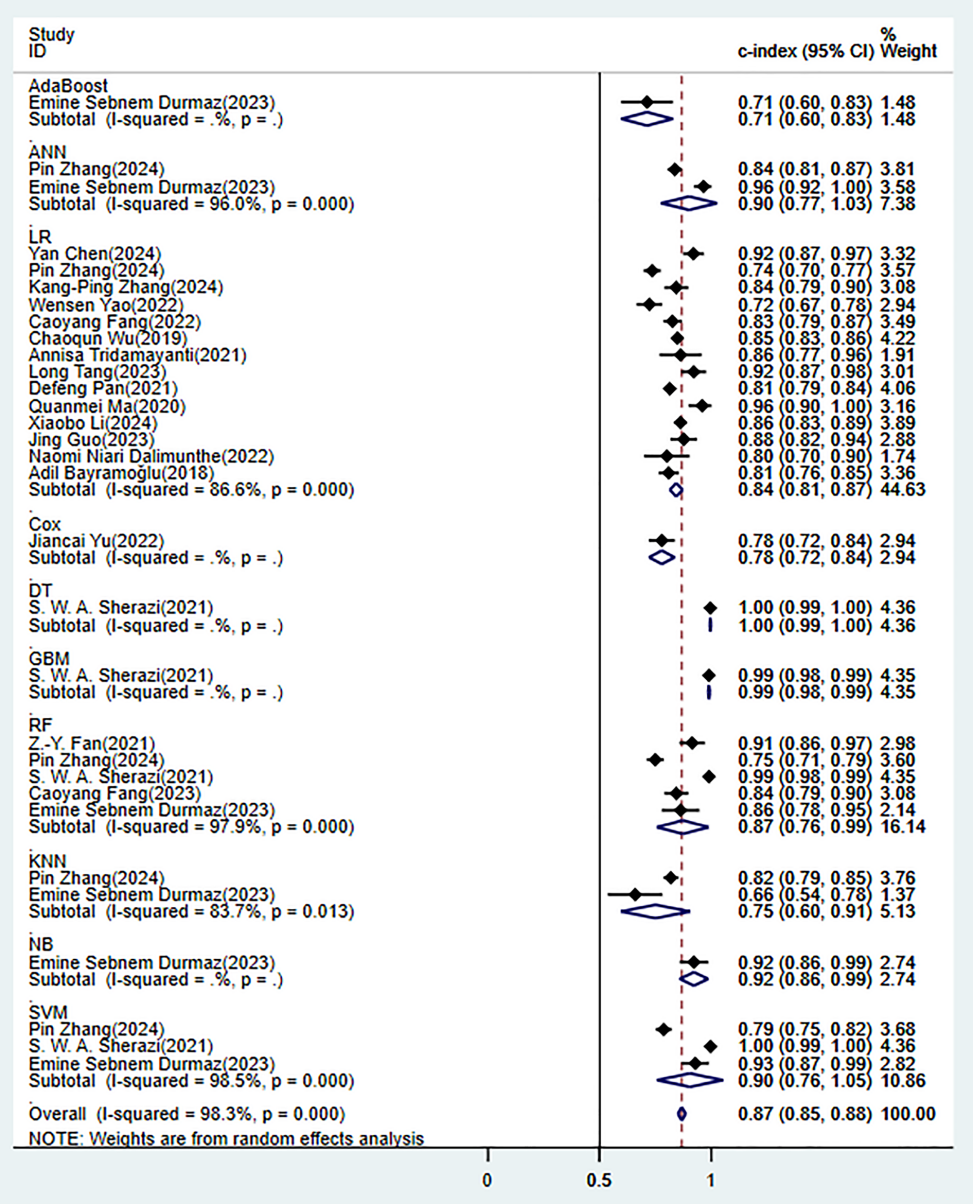
**Random effects model results in the training set**. AdaBoost, adaptive boosting; ANN, artificial neural network; LR, logistic regression; Cox, cox proportional-hazards model; DT, decision tree; GBM, gradient boosting machine; RF, random forest; KNN, k-nearest neighbors; NB, naïve bayes; SVM, support vector machine.

**Fig. 4.**
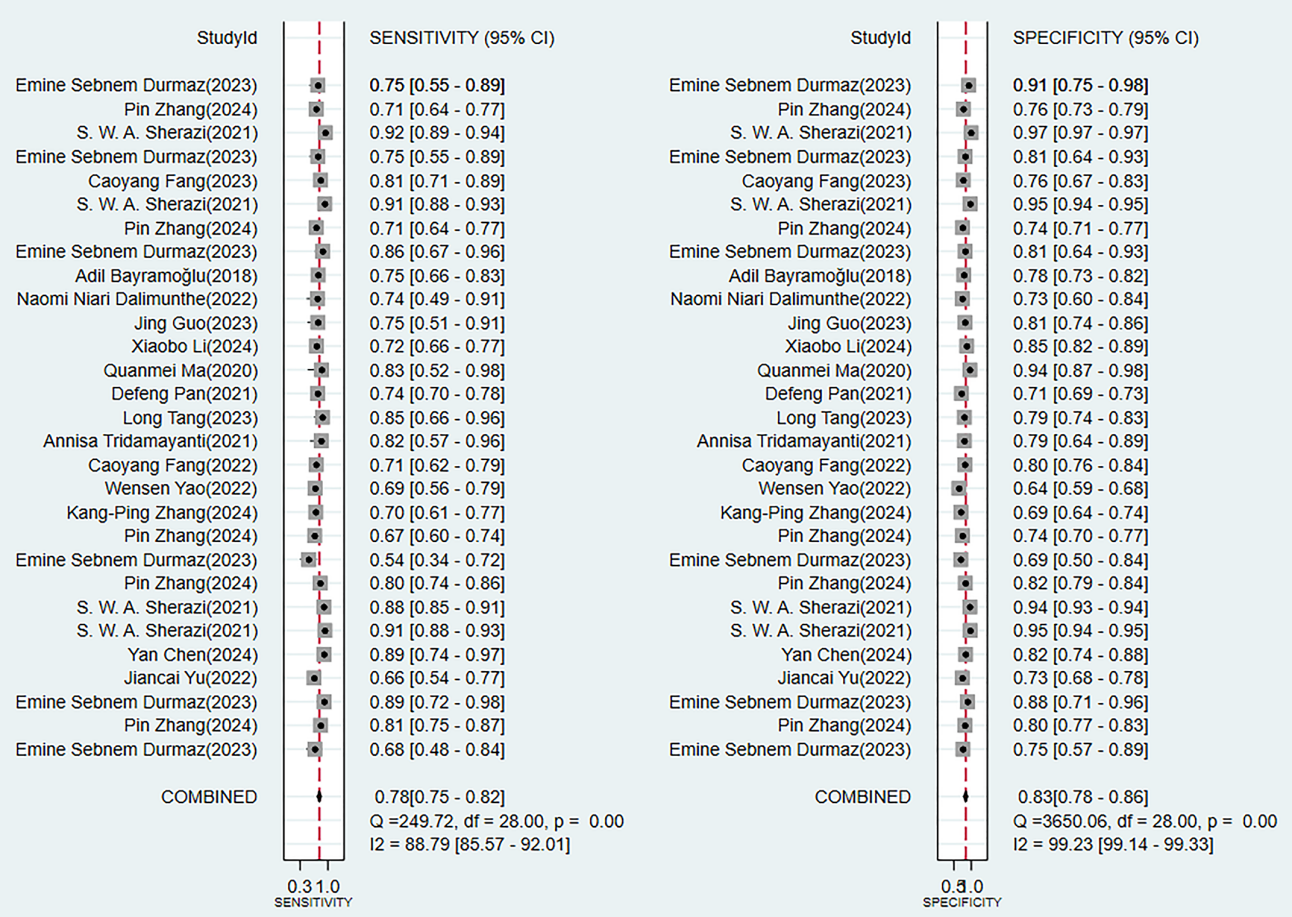
**Sensitivity and specificity analysis results in the training set**.

In the training set, 10 ML models demonstrated excellent predictive performance (Fig. 3). Among them, the performances of alignment diagram based on logistic regression was particularly outstanding (**Supplementary Fig. 1**). The results of the comprehensive analysis of sensitivity (SEN) and specificity (SPE) for these models all indicated good diagnostic accuracy (Fig. 4).

**Fig. 5.**
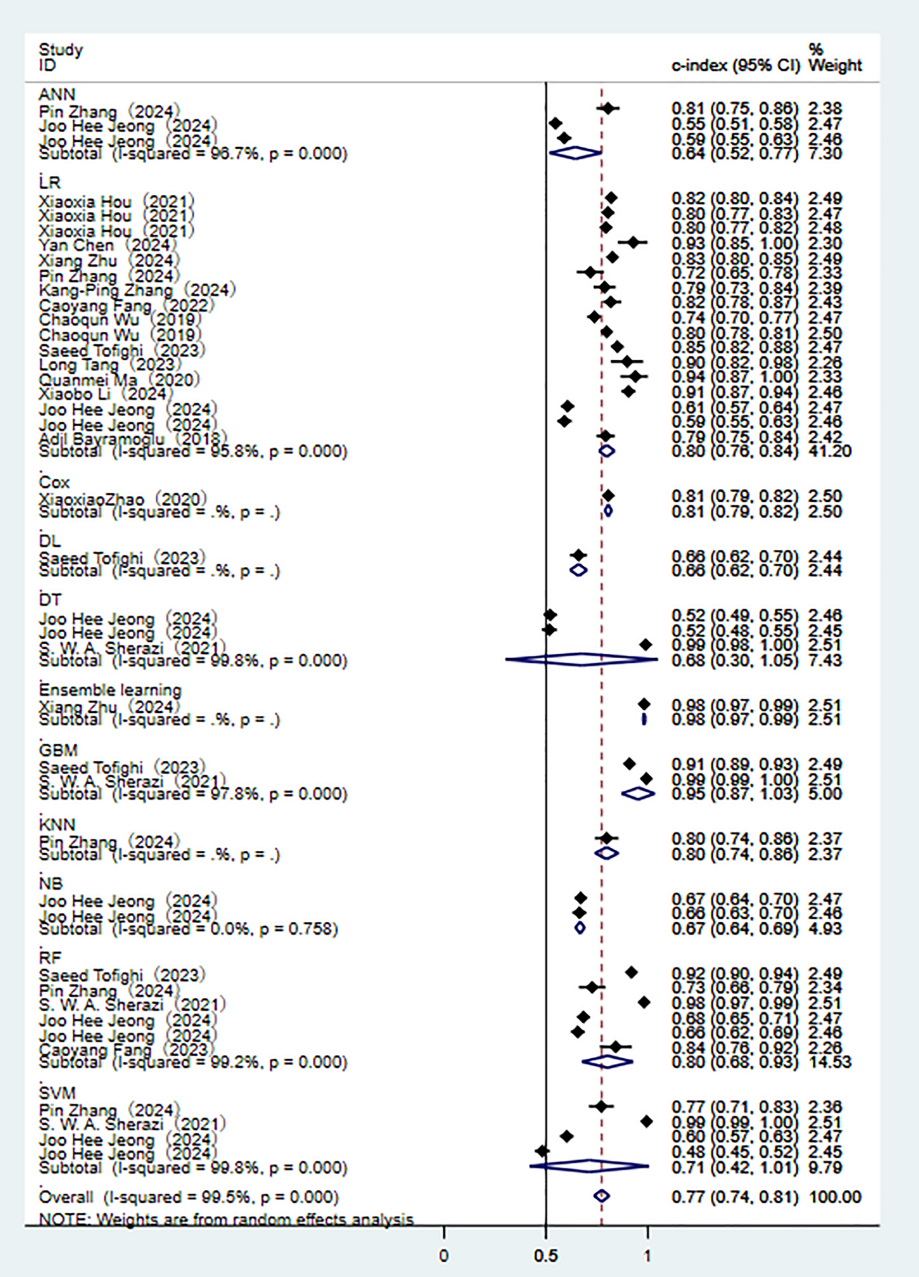
**Random effects model results in the validation set**.

**Fig. 6.**
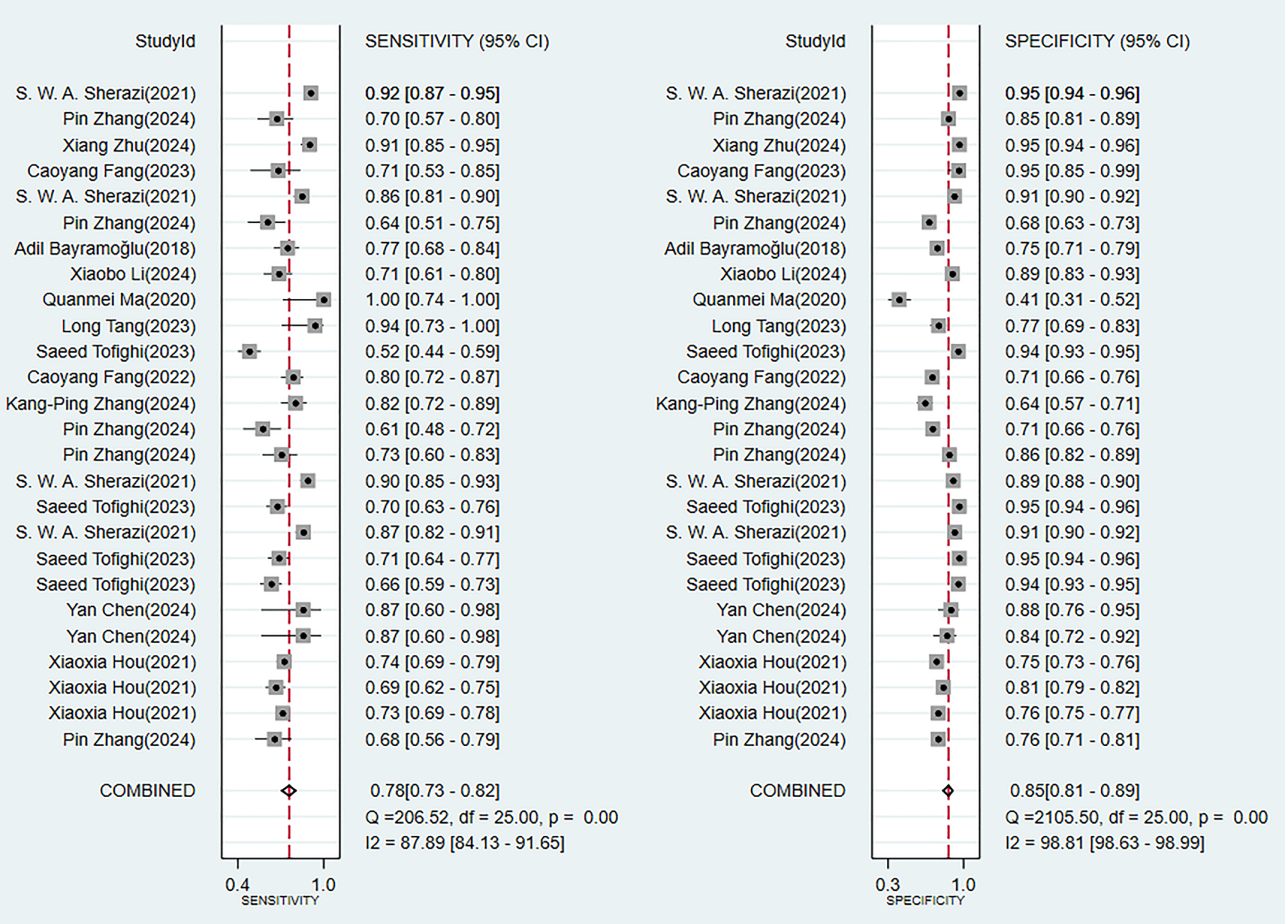
**Analysis results of sensitivity and specificity in the validation set**.

In the validation set (these results were decisive for evaluating the clinical applicability of the models), 11 prediction models demonstrated robust discriminatory performance (Fig. 5). It should be noted that the nomogram model based on logistic regression demonstrated the excellent generalization ability in external validation (**Supplementary Fig. 2**). The consistency of its SEN and SPE indicators (Fig. 6) further confirmed the reliable predictive value of this model in the real-world clinical scenarios.

**Fig. 7.**
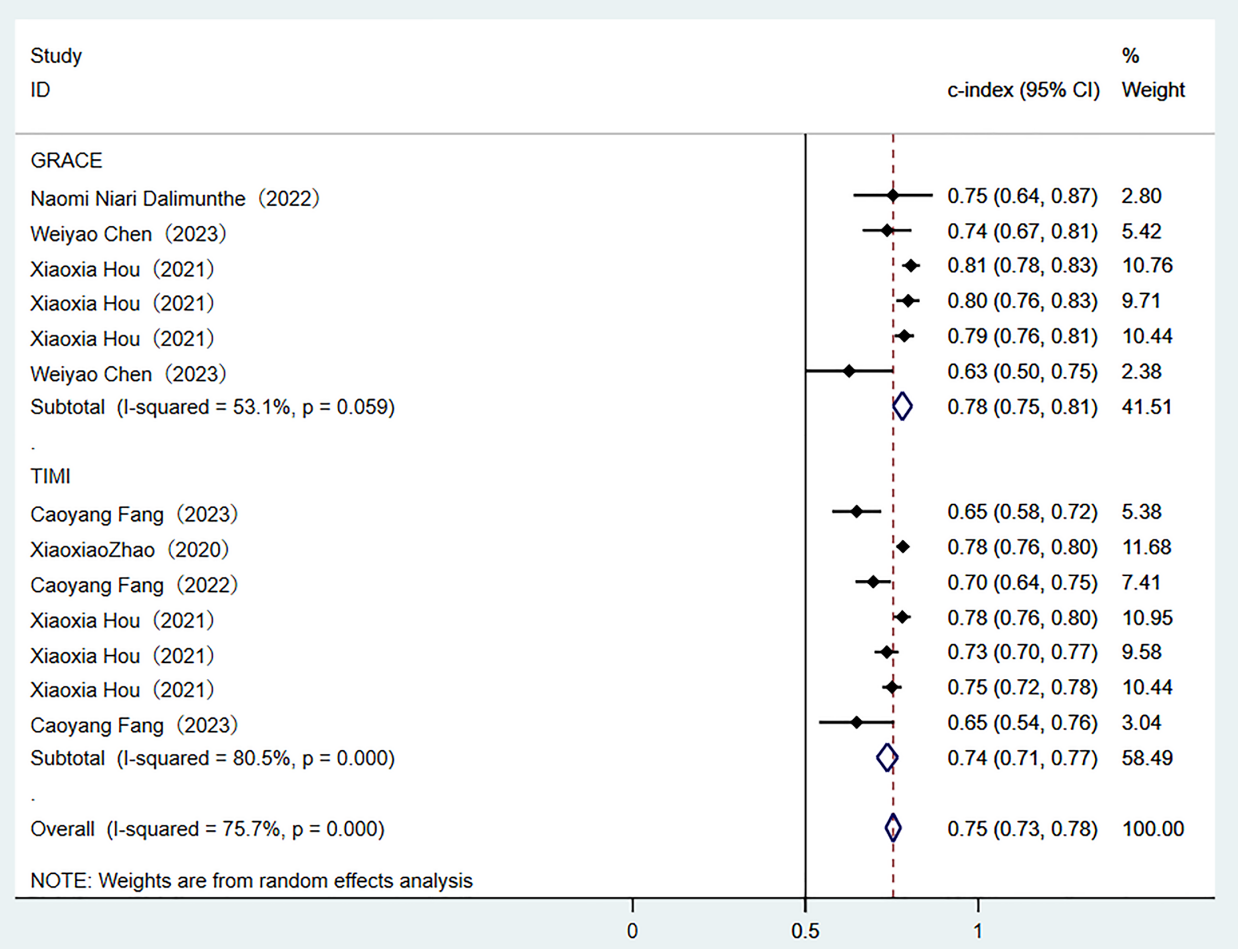
**Random-effects model results of scoring tools**. GRACE, global registry of acute coronary events; TIME, thrombolysis in myocardial infarction.

**Fig. 8.**
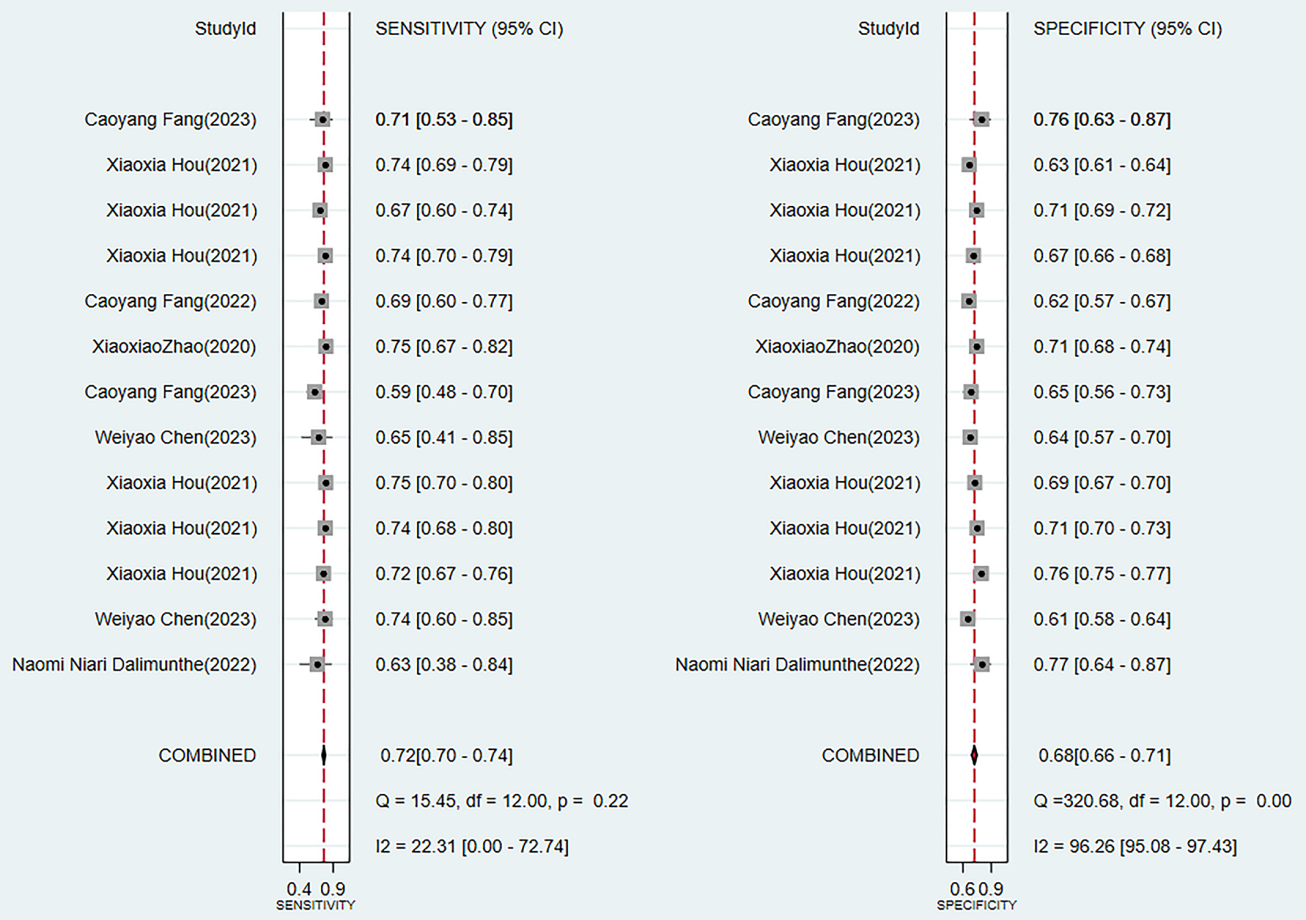
**Sensitivity and specificity analysis results of scoring tools**.

Two traditional scoring tools included in this analysis showed that both the comprehensive predictive efficacy (Fig. 7) and diagnostic accuracy indicators (SEN and SPE data as shown in Fig. 8) were lower than those of the ML models.

3.4.1.2 Reporting Bias

The logistic regression-based prediction nomogram revealed no publication bias. In the training set and the validation set, the probability of the Egger’s test was 0.923 and 0.746, respectively (**Supplementary Figs. 3,4**).

#### 3.4.2 Post-PCI Patients

3.4.2.1 Synthesized Results

In the training set, six models reported the C-index indicator. The analysis of the random-effects model demonstrated good overall predictive performance (**Supplementary Fig. 5**), among which the model based on the logistic regression nomogram showed the most prominent performance (**Supplementary Fig. 6**). The results of the comprehensive analysis of SEN and SPE of these models were shown in detail in **Supplementary Fig. 7**.

The analysis of the validation set (these results of which are of core significance for evaluating the clinical transformation value of the models) showed that the 11 prediction models generally maintained good discriminatory performance (**Supplementary Fig. 8**). It is particularly important to note that the nomogram model based on logistic regression demonstrated excellent generalization performance in independent validation (**Supplementary Fig. 9**). The consistency of its SEN and SPE indicators (**Supplementary Fig. 10**) provides the crucial evidence for the reliable application of the model in real-world medical settings.

3.4.2.2 Reporting Bias

In the validation set. The logistic regression-based prediction nomogram revealed no publication bias. The probability of Egger’s test was 0.417 (**Supplementary Fig. 11**).

#### 3.4.3 Characteristics of Model

Through a systematic evaluation of the training set and the validation set (**Supplementary Figs. 3–8**), the study found that there were significant performance differences among different prediction models. Among the ML models, the best clinical applicability was demonstrated in the logistic regression. The C-index in the validation set remained stable at 0.80 (95% CI 0.76–0.84), there was a good balance between SEN and SPE (**Supplementary Figs. 2,9**). However, its ability to model non-linear relationships was limited. Although the random forest achieved the highest discriminatory performance in the training set (C-index 0.87), there were significant performance fluctuations in the validation set (I^2^ = 99.2%) and the largest attenuation range (ΔC-index = 0.10), which suggested the overfitting risk. The deep learning model showed extremely high SPE (0.95–0.97) in specific scenarios, while it was restricted by data requirements and interpretability. Compared with traditional scoring tools, the overall C-index of ML models increased by 12–15%. Although the GRACE score had relatively good stability (I^2^ = 53.1%), its comprehensive discriminative ability was low (C-index 0.78). The SEN of the TIMI score reached 0.72, while its SPE was significantly lower than that of the ML models (0.68 vs 0.83). It is noteworthy that all models exhibited performance degradation in the validation set, highlighting the necessity of external validation (the complete data was shown in Figs. 3,4,5,6,7,8 and **Supplementary Figs. 1–10**).

## 4. Discussion

### 4.1 Summary of the Main Findings

Our review demonstrated that using ML models to predict the incidence of MACE in MI patients is a feasible approach, with favorable predictive accuracy in the validation sets. Analysis using the random-effects model yielded a pooled estimate in the validation sets yielded a C-index of 0.77 (95% CI 0.74–0.81), with SEN and SPE being 0.75 (95% CI 0.67–0.81) and 0.84 (95% CI 0.75–0.90), respectively. Logistic regression-based nomograms had a pooled SEN of 0.76 (95% CI 0.63–0.86) and SPE of 0.72 (95% CI 0.58–0.83).

### 4.2 Comparison With Previous Reviews

Research has also attempted to explore the early prediction of MACEs in MI patients and has provided certain evidence. For instance, in the study performed by Zhao Xiao* et al*. [45], the myocardial salvage index (MSI), quantified through cardiac magnetic resonance (CMR) imaging, was evaluated for predicting MACEs in patients with ST-segment elevation myocardial infarction (STEMI), showing a pooled MSI (95% CI) of 44% (39%–49%) and a pooled MACE incidence (95% CI) of 10% (7%–14%). Additionally, Gongming Luo* et al*. [46] investigated the predictive performance of fragmented QRS for MACE risk in MI patients, showing an in-hospital MACE odds ratio (OR) of 2.48 (95% CI 1.62–3.80; *p* < 0.0001) and a long-term MACE OR of 3.81 (95% CI 2.21–6.57; *p* < 0.00001). Sameh M Hakim* et al*. [47] reviewed the predictive performance of new-onset ST-segment and T-wave changes for MACEs in MI patients, demonstrating a relatively strong predictive performance (C-index: 0.85; 95% CI 0.83–0.90), with SEN of 0.61 (95% CI 0.55–0.67) and SPE of 0.75 (95% CI 0.72–0.78). In comparison, the performance of ML models in predicting MACEs, as found in this review, showed a C-index of 0.77 (95% CI 0.74–0.81), with SEN and SPE being 0.75 (95% CI 0.67–0.81) and 0.84 (95% CI 0.75–0.90), respectively. The synthesized results from ML models did not outperform conventional methods, including CMR, fragmented QRS, and new-onset ST-segment and T-wave changes. Therefore, further improving the performance of ML models should be a focus of future research.

Additionally, in the study by Danielle Louis E. Villanueva* et al*. [48], the risk of MACEs in post-PCI patients was discussed, as well as the predictive performance of these models for that risk. However, PCI is not just limited to MI patients but is also applicable to patients with unstable angina and chronic coronary syndromes. This study did not address the predictive value of MACE risk in different populations. When constructing models, predictors may differ across populations, which can influence the predictive performance of ML. In our study, the predictive performance of MACE risk in MI patients undergoing PCI was explored. Analysis using the random-effects model to create a pooled estimate for this patient group yielded a C-index of 0.73 (95% CI 0.66–0.79), with SEN and SPE being 0.75 (95% CI 0.67–0.81) and 0.84 (95% CI 0.75–0.90), respectively.

Additionally, some researchers have also explored predictors for the occurrence of MACEs in MI patients. Guoxia Dong* et al*. [49] found that a higher preoperative platelet-to-lymphocyte ratio (PLR) was independently associated with enhanced risk of MACEs (risk ratio [RR]: 1.76, 95% CI 1.39–2.22). In the study by Singh-Baniya Bibek* et al*. [50], elevated preoperative C-reactive protein level was shown to potentially increase the risk of MACEs in a PCI-treated MI population, with a pooled RR of 1.97 (95% CI 1.65–2.35). Moreover, Frederik T W Groenland* et al*. [51] reviewed that intravascular ultrasound-guided PCI may reduce the risk of MACEs in MI patients (RR: 0.86, 95% CI 0.74–0.99). Jun Chen* et al*. [52] also found that high levels of serum cystatin C may increase the risk of MACEs after coronary revascularization in an AMI population (RR: 2.52, 95% CI 1.63–3.89). Furthermore, Jiacheng Rong* et al*. [53] discussed that elevated serum uric acid levels may increase the risk of MACEs in MI patients. In our study, predictors identified for assessing MACE risk included serum VCAM-1, ICAM-1, GDF-15, interleukin-1β, interleukin-17, tumor necrosis factor-alpha (TNF-α), and serum YKL-40, among others.

### 4.3 Differences in Prediction Performance Among Different Machine Learning Models

It was found that in this study, the complex ML models (such as random forests and deep learning) demonstrated excellent performance in the training set, while their performance on the validation set was significantly inferior to that of logistic regression (C-index: 0.80 vs 0.76). This difference mainly stems from three aspects: Firstly, the advantage of logistic regression lies in its ability to incorporate both continuous and categorical independent variables simultaneously, meaning it can adjust for multiple predictive factors. This characteristic makes logistic regression particularly practical in the analysis of observational data [54]; Secondly, the characteristics of medical data (limited sample size, strong linear correlation, and mainly structured variables) are more compatible with the modeling assumptions of logistic regression. In contrast, complex models are prone to overfitting when the data is insufficient.

Despite the rapid development of ML technologies, logistic regression remains irreplaceable in clinical prediction: (1) Longitudinal validation studies consistently demonstrate its superior robustness [55, 56]; (2) It has outstanding computational efficiency, with relatively short training time, low hardware requirements, and less time-consuming result interpretation; (3) It is highly compatible with the existing medical system. Traditional scores such as GRACE/TIMI were based on the same framework, which facilitates the integration and improvement of clinical decision-making.

### 4.4 Advantages and Limitations of the Study

Although our study provided the first evidence-based evaluation of ML methods for predicting the risk of MACEs in MI patients, limitations should also be discussed. Firstly, the quality of the studies varies widely. The PROBAST assessment showed that most of the studies have flaws in their analytical methods, and only a small portion of them report the calibration index. Secondly, there is significant heterogeneity among the models. The differences in the prediction time points, the definitions of MACE, and the number of variables are quite prominent, which may impose certain limitations on the interpretation of the results. In addition, the insufficient sample size of the studies leads to an increased risk of overfitting for complex models and the exacerbated performance fluctuations of studies with small sample sizes. Among the validation methods, only a few studies conduct external validation, there are no reports on the clinical implementation effects, with the insufficient transparency of the reports. These limitations suggest that the performance of the existing models may have been overestimated. From the perspective of the research results, machine learning is superior to the traditional GRACE and TIMI scoring tools. Future research should focus on constructing a standardized validation framework, carrying out three-stage validation (temporal validation, geographical validation, and prospective clinical trials) through multi-center collaboration, and establishing a unified core indicator set for MACE (at least include cardiogenic death, reinfarction, and revascularization and other major endpoints), to further optimize the existing scoring tools.

## 5. Conclusion

This systematic review demonstrated that constructing ML models to predict the onset of MACEs in MI patients is a feasible approach. ML models can accurately predict the onset of MACEs in MI patients and have exhibited favorable discriminatory abilities in retrospective cohorts. The results of the study can provide a basis for auxiliary decision-making and help early identification of high-risk groups. We found that predictors associated with model performance have significant clinical implications for predicting MACEs in MI patients. Although individual models often outperform conventional scoring tools, larger sample size studies are needed to further assess their clinical utility in MI patients. In the existing studies, during the model validation process, the applicability of the model to special populations has not been taken into account. Therefore, in future ML, we should verify the effectiveness of ML in specific subgroups to achieve an improvement in the performance of the model.

## Data Availability

All data generated or analysed during this study are included in this published article and its supplementary information files.

## Abbreviations

ACS, acute coronary syndrome; MI, myocardial infarction; MACEs, major adverse cardiovascular events; PCI, percutaneous coronary intervention; PROBAST, prediction model risk of bias assessment tool; SEN, sensitivity; SPE, specificity; GRACE, Global Registry of Acute Coronary Events; KAMIR, Korea Acute Myocardial Infarction Registry; TIMI, Thrombolysis in Myocardial Infarction; GDF-15, growth differentiation factor 15; CI, confidence interval; SD, standard error; MSI, myocardial salvage index; CMR, cardiac magnetic resonance; STEMI, ST-segment elevation myocardial infarction; PLR, platelet-to-lymphocyte ratio; TNF-α, tumor necrosis factor-alpha; ICAM-1, intercellular cell adhesion molecule-1; VCAM-1, vascular cell adhesion molecule 1.

## Supplementary Material

Supplementary material associated with this article can be found, in the online version, at https://doi.org/10.31083/RCM37224.
