# Supplementary material for: Accuracy of Machine Learning Models for Early Prediction of Major Cardiovascular Events Post Myocardial Infarction: A Systematic Review and Meta-Analysis
Source: Rev Cardiovasc Med. 2025 Jun 17;26(6):37224. doi: 10.31083/RCM37224 (PMC12230836; doi:10.31083/RCM37224)
Supplement: Supplementary file 1 [file 2153-8174-26-6-37224-s1.zip › Supplementary Figs.docx]

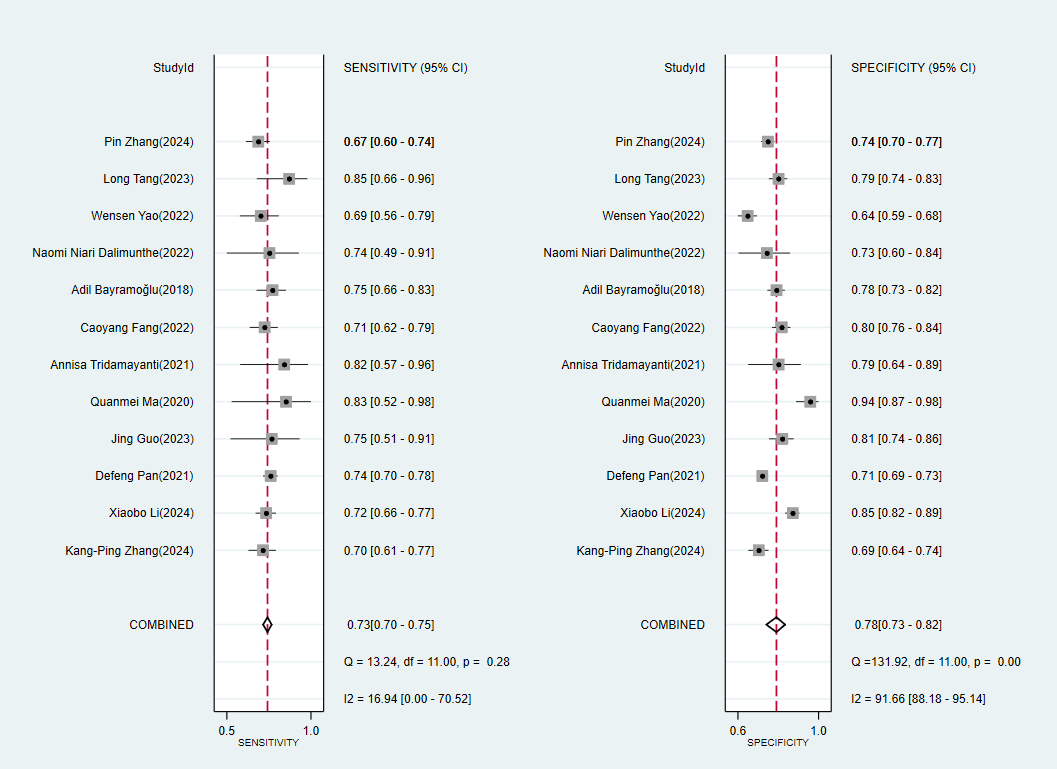


Supplementary Fig. 1 Logistic regression sensitivity and specificity analysis results


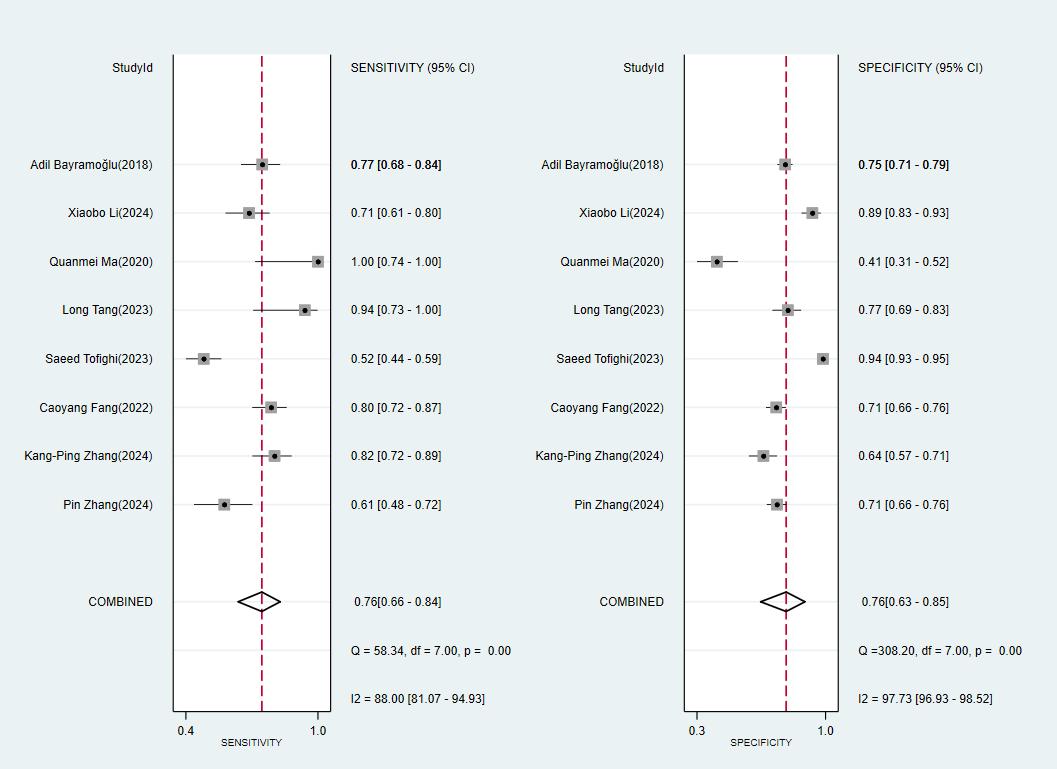


Supplementary Fig. 2 Logistic regression sensitivity and specificity analysis results in the validation set


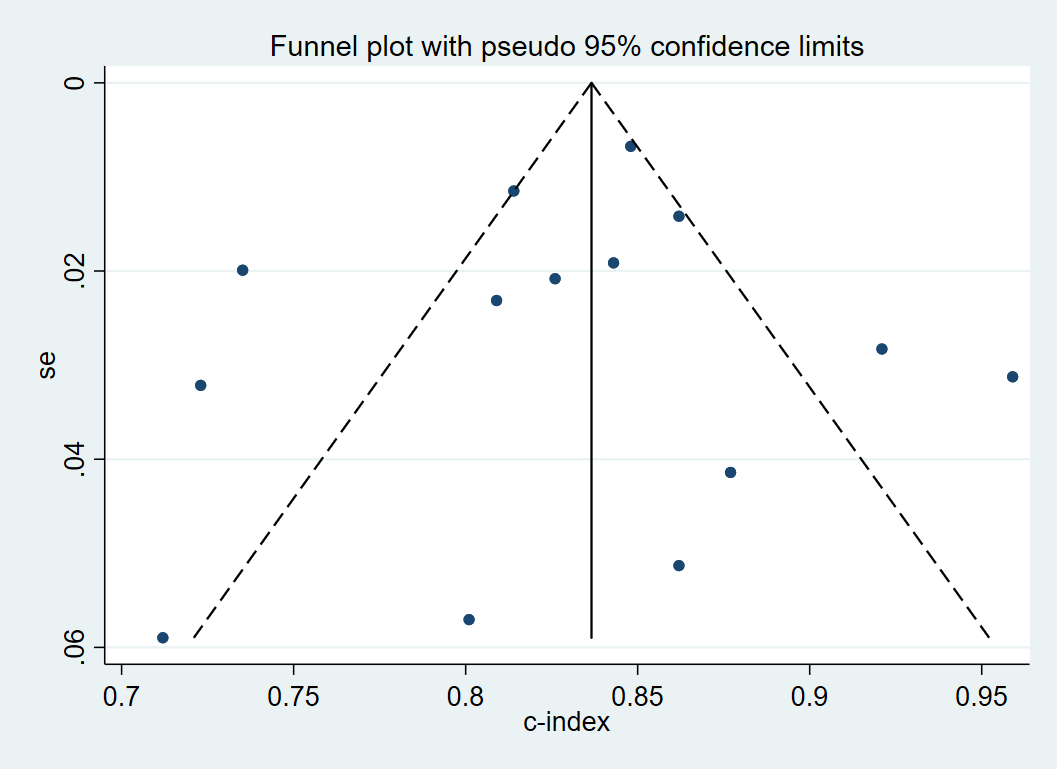


Supplementary Fig. 3 Funnel plot for the C-index of logistic regression in the training set


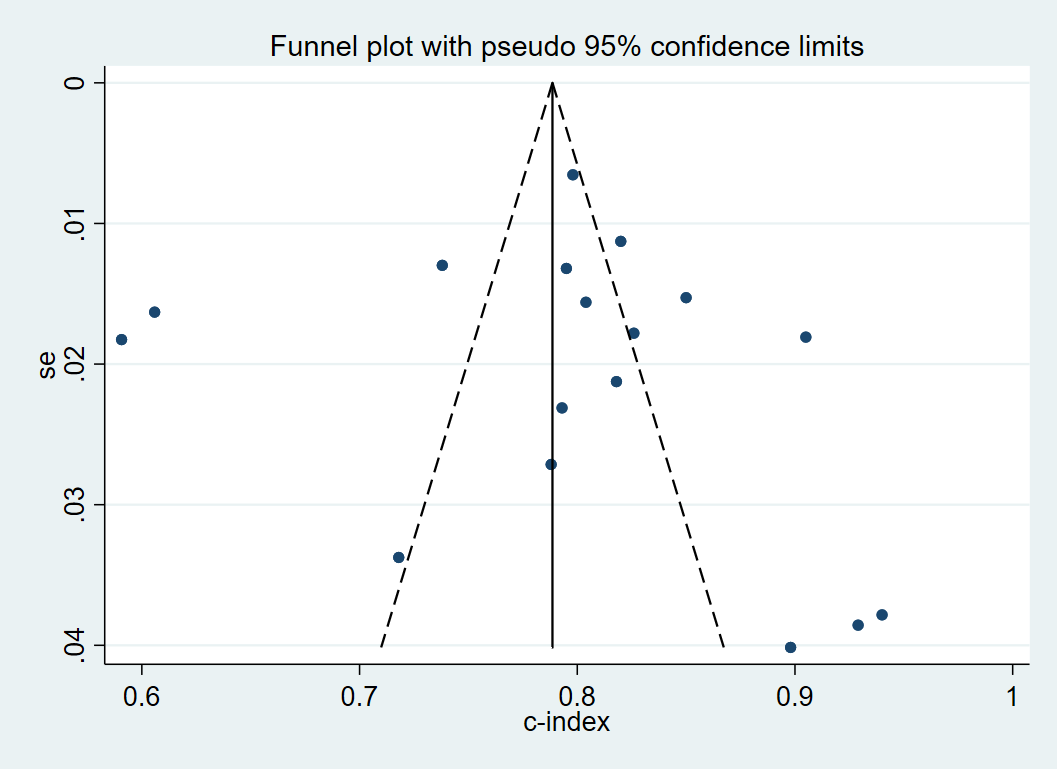


Supplementary Fig. 4 Funnel plot for the C-index of logistic regression in the validation set


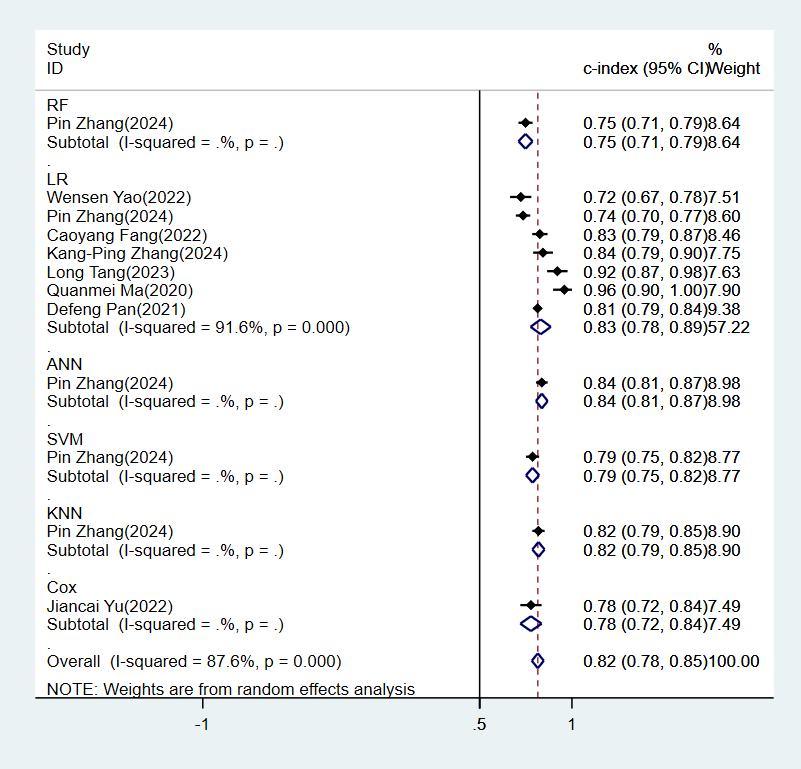


Supplementary Fig. 5 Post-PCI random effects model results in the training set


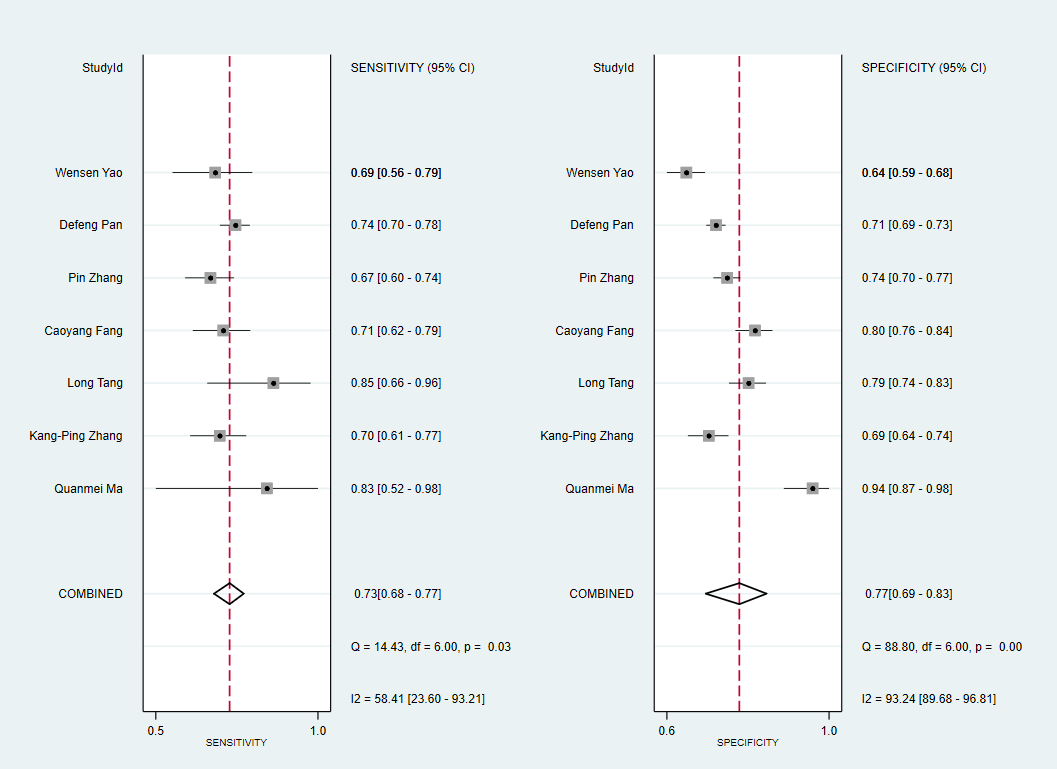


Supplementary Fig. 6 Post-PCI sensitivity and specificity analysis results of logistic regression in the training set


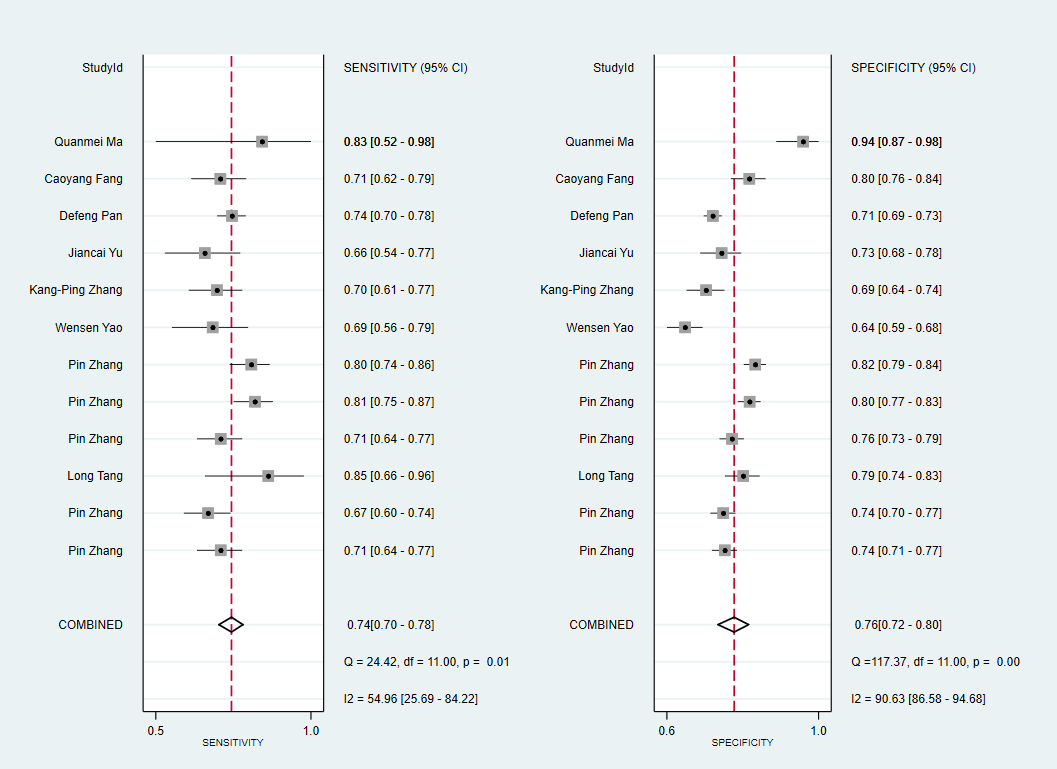


Supplementary Fig. 7 Post-PCI sensitivity and specificity analysis results in the training set


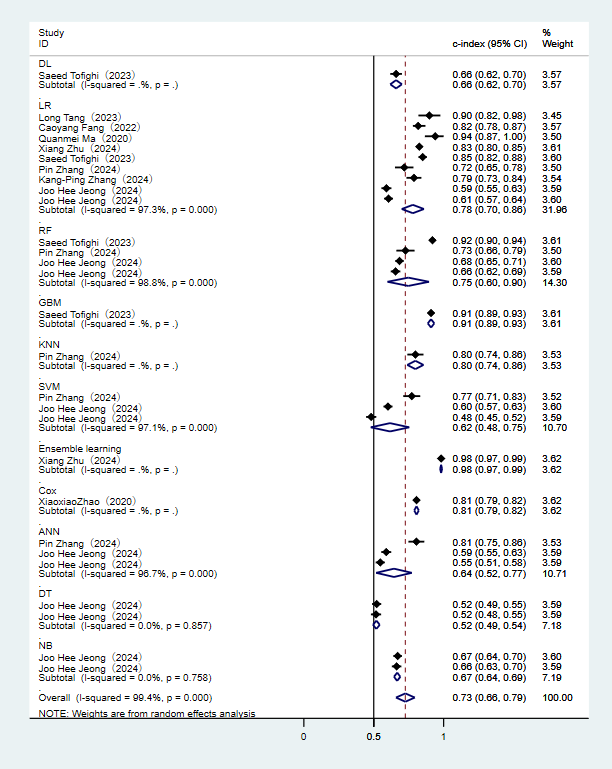


Supplementary Fig. 8 Post-PCI random effects model results in the validation set


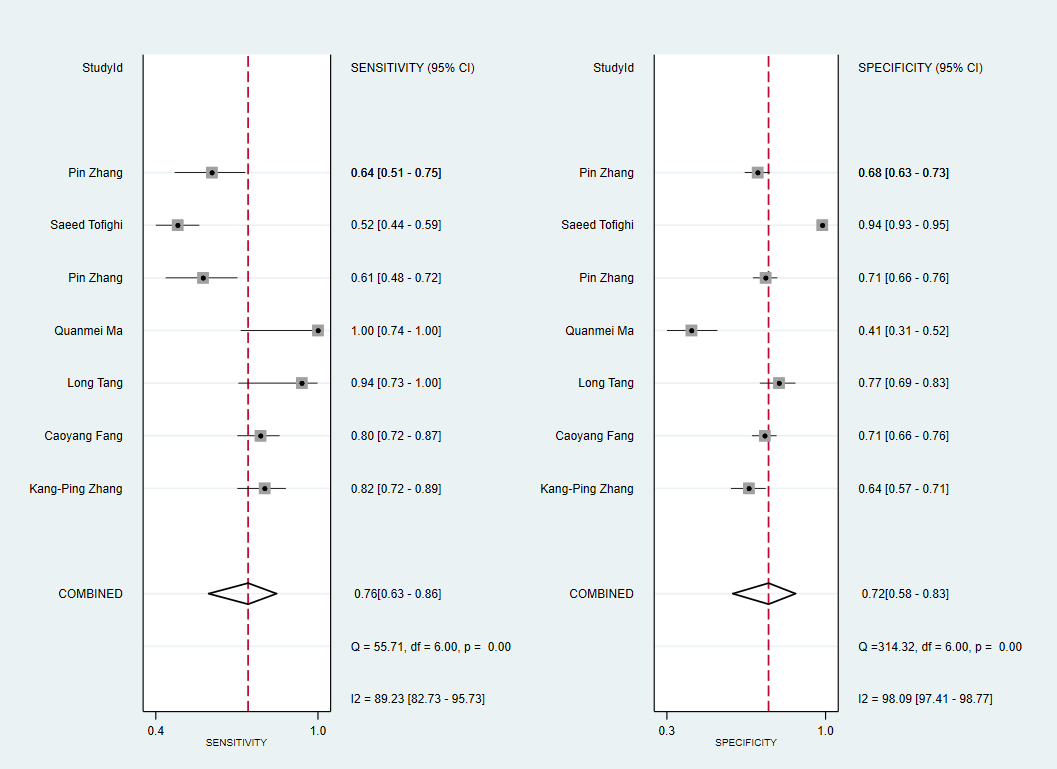


Supplementary Fig. 9 Post-PCI sensitivity and specificity analysis results of logistic regression in the validation set


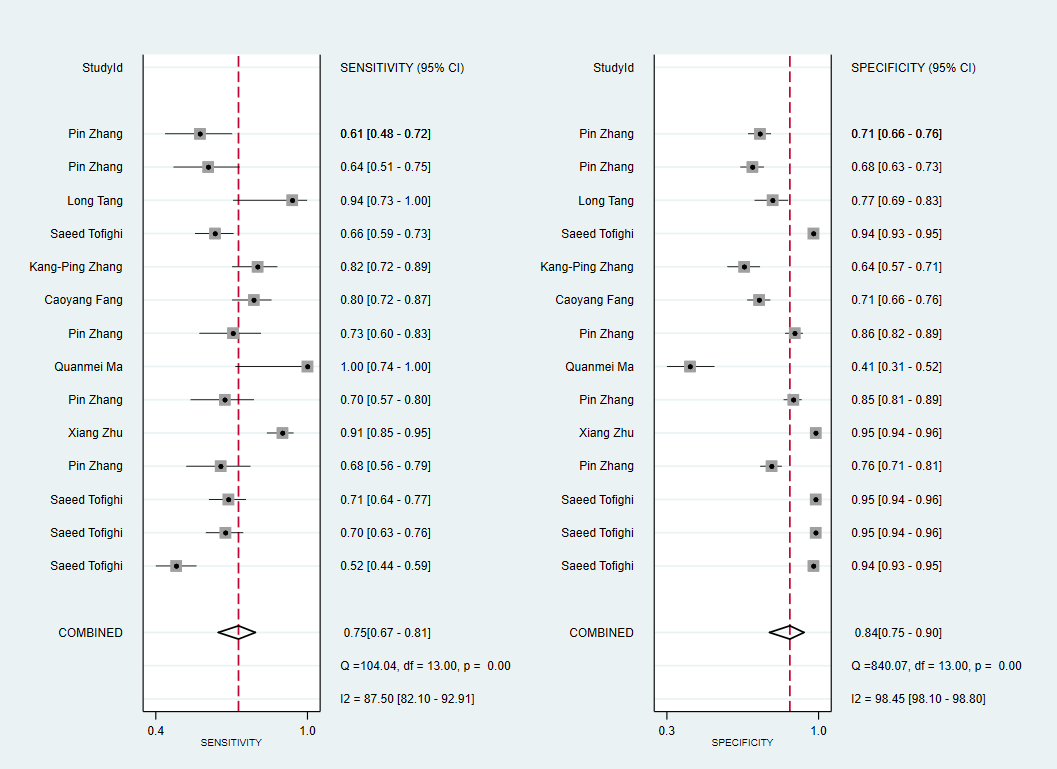


Supplementary Fig. 10 Post-PCI sensitivity and specificity analysis results in the validation set


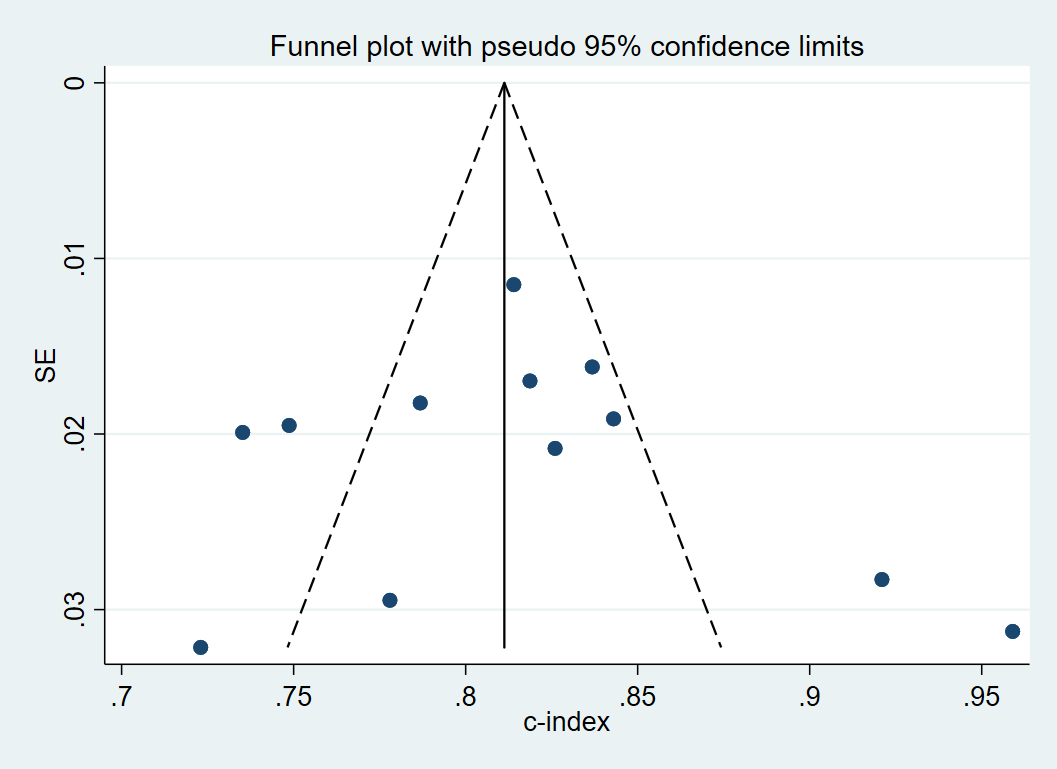


Supplementary Fig. 11 Funnel plot for the C-index of logistic regression predicting post-PCI outcomes
