# Supplementary material for: Accuracy of Machine Learning Models for Early Prediction of Major Cardiovascular Events Post Myocardial Infarction: A Systematic Review and Meta-Analysis
Source: Rev Cardiovasc Med. 2025 Jun 17;26(6):37224. doi: 10.31083/RCM37224 (PMC12230836; doi:10.31083/RCM37224)
Supplement: Supplementary file 1 [file 2153-8174-26-6-37224-s1.zip › Supplementary Table 1.docx]

# Table S2 Literature search strategy

**1.Pubmed**

| Search number | Query | Results |
| --- | --- | --- |
| #1 | Myocardial Infarction[MeSH Terms] | 198,171 |
| #2 | (((((((((((((((((((Myocardial Infarction[Title/Abstract]) OR (Cardiovascular Stroke[Title/Abstract])) OR (Cardiovascular Strokes[Title/Abstract])) OR (Myocardial Infarct[Title/Abstract])) OR (Myocardial Infarcts[Title/Abstract])) OR (Heart Attack[Title/Abstract])) OR (Heart Attacks[Title/Abstract])) OR (MINOCA[Title/Abstract])) OR (NSTEMI[Title/Abstract])) OR (STEMI[Title/Abstract])) OR (cardiac infarct[Title/Abstract])) OR (cardiac infarction[Title/Abstract])) OR (cardial infarct[Title/Abstract])) OR (heart attack[Title/Abstract])) OR (heart infarct[Title/Abstract])) OR (heart infarction[Title/Abstract])) OR (heart micro infarction[Title/Abstract])) OR (heart muscle infarction[Title/Abstract])) OR (myocardium infarction[Title/Abstract])) OR (myocardium infarct[Title/Abstract]) | 242,867 |
| #3 | (Myocardial Infarction[MeSH Terms]) OR ((((((((((((((((((((Myocardial Infarction[Title/Abstract]) OR (Cardiovascular Stroke[Title/Abstract])) OR (Cardiovascular Strokes[Title/Abstract])) OR (Myocardial Infarct[Title/Abstract])) OR (Myocardial Infarcts[Title/Abstract])) OR (Heart Attack[Title/Abstract])) OR (Heart Attacks[Title/Abstract])) OR (MINOCA[Title/Abstract])) OR (NSTEMI[Title/Abstract])) OR (STEMI[Title/Abstract])) OR (cardiac infarct[Title/Abstract])) OR (cardiac infarction[Title/Abstract])) OR (cardial infarct[Title/Abstract])) OR (heart attack[Title/Abstract])) OR (heart infarct[Title/Abstract])) OR (heart infarction[Title/Abstract])) OR (heart micro infarction[Title/Abstract])) OR (heart muscle infarction[Title/Abstract])) OR (myocardium infarction[Title/Abstract])) OR (myocardium infarct[Title/Abstract])) | 298,659 |
| #4 | machine learning[MeSH Terms] | 70,367 |
| #5 | (((((((((((((((((((((((((((((machine learning[Title/Abstract]) OR (Transfer Learning[Title/Abstract])) OR (Deep learning[Title/Abstract])) OR (Ensemble Learning[Title/Abstract])) OR (artificial intelligence[Title/Abstract])) OR (random forest[Title/Abstract])) OR (neural network[Title/Abstract])) OR (neural networks[Title/Abstract])) OR (K-Nearest Neighbor[Title/Abstract])) OR (CNN[Title/Abstract])) OR (Support vector machine[Title/Abstract])) OR (SVM[Title/Abstract])) OR (Gradient Boosting Machine[Title/Abstract])) OR (Nomogram[Title/Abstract])) OR (XGBoost[Title/Abstract])) OR (Adaboost[Title/Abstract])) OR (Decision tree[Title/Abstract])) OR (ResNet-50[Title/Abstract])) OR (ResNet[Title/Abstract])) OR (AlexNet[Title/Abstract])) OR (VGGNet[Title/Abstract])) OR (GoogLeNet[Title/Abstract])) OR (Naive Bayesian[Title/Abstract])) OR (Multilayer perceptron[Title/Abstract])) OR (Bayesian network[Title/Abstract])) OR (Radiomics[Title/Abstract])) OR (Radiomic[Title/Abstract])) OR (Prediction model[Title/Abstract])) OR (Risk model[Title/Abstract])) OR (Risk score[Title/Abstract]) | 385,719 |
| #6 | (machine learning[MeSH Terms]) OR ((((((((((((((((((((((((((((((machine learning[Title/Abstract]) OR (Transfer Learning[Title/Abstract])) OR (Deep learning[Title/Abstract])) OR (Ensemble Learning[Title/Abstract])) OR (artificial intelligence[Title/Abstract])) OR (random forest[Title/Abstract])) OR (neural network[Title/Abstract])) OR (neural networks[Title/Abstract])) OR (K-Nearest Neighbor[Title/Abstract])) OR (CNN[Title/Abstract])) OR (Support vector machine[Title/Abstract])) OR (SVM[Title/Abstract])) OR (Gradient Boosting Machine[Title/Abstract])) OR (Nomogram[Title/Abstract])) OR (XGBoost[Title/Abstract])) OR (Adaboost[Title/Abstract])) OR (Decision tree[Title/Abstract])) OR (ResNet-50[Title/Abstract])) OR (ResNet[Title/Abstract])) OR (AlexNet[Title/Abstract])) OR (VGGNet[Title/Abstract])) OR (GoogLeNet[Title/Abstract])) OR (Naive Bayesian[Title/Abstract])) OR (Multilayer perceptron[Title/Abstract])) OR (Bayesian network[Title/Abstract])) OR (Radiomics[Title/Abstract])) OR (Radiomic[Title/Abstract])) OR (Prediction model[Title/Abstract])) OR (Risk model[Title/Abstract])) OR (Risk score[Title/Abstract])) | 391,084 |
| #7 | (((((((Major Adverse Cardiac Events[Title/Abstract]) OR (Major Adverse Cardiac Event[Title/Abstract])) OR (major cardiac adverse event[Title/Abstract])) OR (major cardiac adverse events[Title/Abstract])) OR (MACE[Title/Abstract])) OR (MACEs[Title/Abstract])) OR (major adverse cardiovascular events[Title/Abstract])) OR (major adverse cardiovascular event[Title/Abstract]) | 21042 |
| #8 | (((Myocardial Infarction[MeSH Terms]) OR ((((((((((((((((((((Myocardial Infarction[Title/Abstract]) OR (Cardiovascular Stroke[Title/Abstract])) OR (Cardiovascular Strokes[Title/Abstract])) OR (Myocardial Infarct[Title/Abstract])) OR (Myocardial Infarcts[Title/Abstract])) OR (Heart Attack[Title/Abstract])) OR (Heart Attacks[Title/Abstract])) OR (MINOCA[Title/Abstract])) OR (NSTEMI[Title/Abstract])) OR (STEMI[Title/Abstract])) OR (cardiac infarct[Title/Abstract])) OR (cardiac infarction[Title/Abstract])) OR (cardial infarct[Title/Abstract])) OR (heart attack[Title/Abstract])) OR (heart infarct[Title/Abstract])) OR (heart infarction[Title/Abstract])) OR (heart micro infarction[Title/Abstract])) OR (heart muscle infarction[Title/Abstract])) OR (myocardium infarction[Title/Abstract])) OR (myocardium infarct[Title/Abstract]))) AND ((machine learning[MeSH Terms]) OR ((((((((((((((((((((((((((((((machine learning[Title/Abstract]) OR (Transfer Learning[Title/Abstract])) OR (Deep learning[Title/Abstract])) OR (Ensemble Learning[Title/Abstract])) OR (artificial intelligence[Title/Abstract])) OR (random forest[Title/Abstract])) OR (neural network[Title/Abstract])) OR (neural networks[Title/Abstract])) OR (K-Nearest Neighbor[Title/Abstract])) OR (CNN[Title/Abstract])) OR (Support vector machine[Title/Abstract])) OR (SVM[Title/Abstract])) OR (Gradient Boosting Machine[Title/Abstract])) OR (Nomogram[Title/Abstract])) OR (XGBoost[Title/Abstract])) OR (Adaboost[Title/Abstract])) OR (Decision tree[Title/Abstract])) OR (ResNet-50[Title/Abstract])) OR (ResNet[Title/Abstract])) OR (AlexNet[Title/Abstract])) OR (VGGNet[Title/Abstract])) OR (GoogLeNet[Title/Abstract])) OR (Naive Bayesian[Title/Abstract])) OR (Multilayer perceptron[Title/Abstract])) OR (Bayesian network[Title/Abstract])) OR (Radiomics[Title/Abstract])) OR (Radiomic[Title/Abstract])) OR (Prediction model[Title/Abstract])) OR (Risk model[Title/Abstract])) OR (Risk score[Title/Abstract])))) AND ((((((((Major Adverse Cardiac Events[Title/Abstract]) OR (Major Adverse Cardiac Event[Title/Abstract])) OR (major cardiac adverse event[Title/Abstract])) OR (major cardiac adverse events[Title/Abstract])) OR (MACE[Title/Abstract])) OR (MACEs[Title/Abstract])) OR (major adverse cardiovascular events[Title/Abstract])) OR (major adverse cardiovascular event[Title/Abstract])) | 522 |

**2.Cochrane**

| Search number | Query | Results |
| --- | --- | --- |
| #1 | MeSH descriptor: [Myocardial Infarction] explode all trees | 15663 |
| #2 | (Myocardial Infarction):ti,ab,kw OR (Myocardial Infarctions):ti,ab,kw OR (Cardiovascular Stroke):ti,ab,kw OR (Cardiovascular Strokes):ti,ab,kw OR (Myocardial Infarct):ti,ab,kw | 44275 |
| #3 | (Myocardial Infarcts):ti,ab,kw OR (Heart Attack):ti,ab,kw OR (Heart Attacks):ti,ab,kw OR (MINOCA):ti,ab,kw OR (NSTEMI):ti,ab,kw | 5412 |
| #4 | (STEMI):ti,ab,kw OR (cardiac infarct):ti,ab,kw OR (cardiac infarction):ti,ab,kw OR (cardial infarct):ti,ab,kw OR (heart infarction):ti,ab,kw | 31948 |
| #5 | (heart micro infarction):ti,ab,kw OR (heart muscle infarction):ti,ab,kw OR (myocardium infarction):ti,ab,kw OR (myocardium infarct):ti,ab,kw | 4975 |
| #6 | #1 or #2 or #3 or #4 or #5 | 50515 |
| #7 | MeSH descriptor: [Machine Learning] explode all trees | 986 |
| #8 | (Transfer Learning):ti,ab,kw OR (Deep learning):ti,ab,kw OR (Ensemble Learning):ti,ab,kw OR (artificial intelligence):ti,ab,kw OR (random forest):ti,ab,kw | 5912 |
| #9 | (neural network):ti,ab,kw OR (neural networks):ti,ab,kw OR (K-Nearest Neighbor):ti,ab,kw OR (CNN):ti,ab,kw OR (Support vector machine):ti,ab,kw | 4195 |
| #10 | (SVM):ti,ab,kw OR (Gradient Boosting Machine):ti,ab,kw OR (Nomogram):ti,ab,kw OR (XGBoost):ti,ab,kw OR (Adaboost):ti,ab,kw | 2315 |
| #11 | (Decision tree):ti,ab,kw OR (ResNet-50):ti,ab,kw OR (ResNet):ti,ab,kw OR (AlexNet):ti,ab,kw OR (VGGNet):ti,ab,kw | 1047 |
| #12 | (GoogLeNet):ti,ab,kw OR (Naive Bayesian):ti,ab,kw OR (Multilayer perceptron):ti,ab,kw OR (Bayesian network):ti,ab,kw OR (Radiomics):ti,ab,kw | 1177 |
| #13 | (Radiomic):ti,ab,kw OR (Prediction model):ti,ab,kw OR (Risk model):ti,ab,kw OR (Risk score):ti,ab,kw | 70481 |
| #14 | #7 or #8 or #9 or #10 or #11 or #12 or #13 | 80084 |
| #15 | (Major Adverse Cardiac Events):ti,ab,kw OR (Major Adverse Cardiac Event):ti,ab,kw OR (major cardiac adverse event):ti,ab,kw OR (major cardiac adverse events):ti,ab,kw OR (MACE):ti,ab,kw | 10390 |
| #16 | (MACEs):ti,ab,kw OR (major adverse cardiovascular events):ti,ab,kw OR (major adverse cardiovascular event):ti,ab,kw | 9677 |
| #17 | #6 and #14 and #15 and #16 | 467 |

**3.Embase**

| Search number | Query | Results |
| --- | --- | --- |
| #1 | 'heart infarction'/exp | 485234 |
| #2 | myocardial infarction':ab,ti OR 'myocardial infarctions':ab,ti OR 'cardiovascular stroke':ab,ti OR 'cardiovascular strokes':ab,ti OR 'myocardial infarct':ab,ti OR 'myocardial infarcts':ab,ti OR 'heart attack':ab,ti OR 'heart attacks':ab,ti OR minoca:ab,ti OR nstemi:ab,ti OR stemi:ab,ti OR 'cardiac infarct':ab,ti OR 'cardiac infarction':ab,ti OR 'cardial infarct':ab,ti OR 'heart infarction':ab,ti OR 'heart micro infarction':ab,ti OR 'heart muscle infarction':ab,ti OR 'myocardium infarction':ab,ti OR 'myocardium infarct':ab,ti | 363182 |
| #3 | #1 OR #2 | 536815 |
| #4 | 'machine learning'/exp | 486148 |
| #5 | machine learning':ab,ti OR 'transfer learning':ab,ti OR 'deep learning':ab,ti OR 'ensemble learning':ab,ti OR 'artificial intelligence':ab,ti OR 'random forest':ab,ti OR 'neural network':ab,ti OR 'neural networks':ab,ti OR 'k-nearest neighbor':ab,ti OR cnn:ab,ti OR 'support vector machine':ab,ti OR svm:ab,ti OR 'gradient boosting machine':ab,ti OR nomogram:ab,ti OR xgboost:ab,ti OR adaboost:ab,ti OR 'decision tree':ab,ti OR 'resnet 50':ab,ti OR resnet:ab,ti OR alexnet:ab,ti OR vggnet:ab,ti OR googlenet:ab,ti OR 'naive bayesian':ab,ti OR 'multilayer perceptron':ab,ti OR 'bayesian network':ab,ti OR radiomics:ab,ti OR radiomic:ab,ti OR 'prediction model':ab,ti OR 'risk model':ab,ti OR 'risk score':ab,ti | 456591 |
| #6 | #4 OR #5 | 695302 |
| #7 | 'major adverse cardiac events':ab,ti OR 'major adverse cardiac event':ab,ti OR 'major cardiac adverse event':ab,ti OR 'major cardiac adverse events':ab,ti OR mace:ab,ti OR maces:ab,ti OR 'major adverse cardiovascular events':ab,ti OR 'major adverse cardiovascular event':ab,ti | 40923 |
| #8 | #3 AND #6 AND #7 | 1079 |

**4.Web of science**

| Search number | Query | Results |
| --- | --- | --- |
| #1 | (Myocardial Infarction OR Myocardial Infarctions OR Cardiovascular Stroke OR Cardiovascular Strokes OR Myocardial Infarct OR Myocardial Infarcts OR Heart Attack OR Heart Attacks OR MINOCA OR NSTEMI OR STEMI OR cardiac infarct OR cardiac infarction OR cardial infarct OR heart infarction OR heart micro infarction OR heart muscle infarction OR myocardium infarction OR myocardium infarct) | 424197 |
| #2 | (machine learning OR Transfer Learning OR Deep learning OR Ensemble Learning OR artificial intelligence OR random forest OR neural network OR neural networks OR K-Nearest Neighbor OR CNN OR Support vector machine OR SVM OR Gradient Boosting Machine OR Nomogram OR XGBoost OR Adaboost OR Decision tree OR ResNet-50 OR ResNet OR AlexNet OR VGGNet OR GoogLeNet OR Naive Bayesian OR Multilayer perceptron OR Bayesian network OR Radiomics OR Radiomic OR Prediction model OR Risk model OR Risk score) | 3547161 |
| #3 | (Major Adverse Cardiac Events OR Major Adverse Cardiac Event OR major cardiac adverse event OR major cardiac adverse events OR MACE OR MACEs OR major adverse cardiovascular events OR major adverse cardiovascular) | 34263 |
| #4 | #55 AND #54 AND #53 | 4698 |
