# Supplementary material for: Accuracy of Machine Learning Models for Early Prediction of Major Cardiovascular Events Post Myocardial Infarction: A Systematic Review and Meta-Analysis
Source: Rev Cardiovasc Med. 2025 Jun 17;26(6):37224. doi: 10.31083/RCM37224 (PMC12230836; doi:10.31083/RCM37224)
Supplement: Supplementary file 1 [file 2153-8174-26-6-37224-s1.zip › Supplementary Table 2.docx]

Table S1 Basic characteristics of included studies

| No. |  | First author | Year of publication | Country of author | Patient source | Treatment background | Definition of MACEs | Follow-up duration | Number of MACE cases | Total number of cases | Number of cases in training set | Generation method of the validation set | Number of cases in the validation set | Model type | Modeling variables |
| --- | --- | --- | --- | --- | --- | --- | --- | --- | --- | --- | --- | --- | --- | --- | --- |
| 1 |  | XiaoxiaoZhao | 2020 | China | Single center | PCI | All-causemortality, cardiacmortality, MI recurrence, and stroke (ischemicstroke). | >1 year | 455 | 4103 | 3078 | Random sampling | 1025 | Cox regression | Age, History of diabetes mellitus, Atrial ﬁbrilla-tion, Chronic kidney disease, Coronary artery bypass grafting, The Killip classiﬁcation, Ejecti-on fraction atadmission, The high-sensitivity C-reactiveprotein level, The estimated glomerular ﬁltration rate, The d-dimerlevel, Multivessel le-sions, Culpritvessel. |
| 2 |  | Pin Zhang | 2024 | China | Single center | PCI | Cardiomyopathies, hypertensive heart d-isease, recurrent myocardial infarction, heart failure, sudden cardiac death, re-vascularization, malignant arrhythmia, and stent thrombosis. | 1 year | 252 | 1362 | 953 | 7-fold cross- validation | 409 | Logistic regression | Left ventricular ejection fraction, The number o-f implanted stents, Age, Diabetes, The number of vessels with coronary artery disease, Vascu-lar disease, Brain natriuretic peptide, Glucose, Beta 2 microglobulin, Abnormal Q-wave. |
| 3 |  | Xiang Zhu | 2024 | China | Single center | PCI | Mainly includes cardiac death, myocardial infarction, angina pectoris attack, heart failure, revascularization, malignant arrhythmia, stent thrombosis, etc. | 1 year | 475 | 3880 | 3751 | 5-fold cross- validation | 1249 | Random Forest, Generalized linear model,  Generalized additive model,  Xgboost,  gradient boosting machines,  support vector machine | Hospitalizations, Number of diseased coronar ar-tery, BNP, Killip, Cardiac arrest. |
| 4 |  | Kang-Ping Zhang | 2024 | China | Multicenter | PCI | Cardiac death. Electrocardiogram and Echocardiography were secondary end points that are myocardial reinfarction, life-threatening arrhythmias and acute heart failure. Diagnosis of acute heart failure included the presence of such symptoms as dyspnea, orthopnea, pulmonary rales and pink foamy sputum; NT-proBNP was determined to be >450 ngL for individuals aged below 50 years old (<50), >900 ng/L in those ≥50 but <75 above,1800 ng-L indicating group III (among the malignant arrhythmias were severe sinus bradycardia (≤40 beatsmin), advanced atrioventricular block or complete heart block, ven-tricular tachycardia, ventricular fibrillation and cardiac arrest;with latter being a specific subset of malignant arrhythmia. | NA | NA | NA | 511 | External validation based on multiple centers | 284 | Logistic regression | SBP, Levels of D-dimer, The ratio of HbA1c to ApoA1, Urea concentration. |
| 5 |  | Enfa Zhao | 2020 | China | Registration database | PCI | The composite of cardiac death, clinically driven target lesion revascularisation, recurrent target vessel myocardial infarction, cardiogenic shock, or demonstrated congestive heart failure. The apelin change rate was defined as the level of apelin-12 at 72 hours after PCI compared with that immediately before PCI.The other clinical outcomes were defined in a previous study | 2.5 years | NA | 464 | 324 | Random sampling | 136 | Cox regression | Apelin-12 change rate, Apelin-12 level, Age, Pathological Q wave, Myocardial infarction history, Anterior wall myocardial infarction, Killip’s classification > I, Uric acid, Total cholesterol and the left atrial diameter. |
| 6 |  | Jiancai Yu | 2022 | China | Single center | PCI | Composite of all-­cause death, any myocardial infarction, and any repeat revascularization. | 18 months | NA | 373 | NA | Random sampling | NA | Cox regression | NA |
| 7 |  | Wensen Yao | 2022 | China | Single center | PCI | A composite of cardiac death, myocardial infarction, hospitalization for chest pain or congestive heart failure, late revascularization, or arrhythmia. | 1 year | NA | 526 | NA | NA | NA | Logistic regression | Sex, Age, Hypertension, Diabetes mellitus, CES, Number of diseased vessels, In-hospital VA, Peak cTnI, LVE, AGR, NT-proBNP, Hemoglobin, Fibrinogen. |
| 8 |  | Caoyang Fang | 2022 | China | Single center | PCI | The primary endpoint as cardiac death. Secondary endpoints were myocardial reinfarction, malignant arrhythmia, and acute heart failure. | NA | 127 | 466 | NA | 10-fold cross- validation | NA | Logistic regression | Systolic and diastolic blood pressure at admission, Killip class II-IV, LVEF, urea, NT-ProBNP, IABP. |
| 9 |  | Chaoqun Wu | 2019 | China | Multicenter |  | A composite of first occurrence of all-­ cause death, recurrent AMI or non-­fatal ischaemic stroke during index hospitalisation. | NA | NA | NA | 8010 | External validation | 15078 | Logistic regression | Age, Sex, LVEF, Killip class, SBP, Creatinine, WBC, Heart rate and blood glucose. |
| 10 |  | Annisa Tridamayanti | 2021 | Indonesia | Single center | NA | Presence of death,reinfarction, and nonfatal heart failure events requiring rehospitalization. | 90 days | 17 | 64 | NA | NA | NA | Logistic regression | GRACE score, eGFR, Urea GDF-15, PCI, ß-Blockers |
| 11 |  | Duc Hung Tran | 2023 | Vietnam | Multicenter | PCI | Any major adverse event after the procedure, such as all-cause mortality, re-infarction, in-stent thrombosis, stroke, re-intervention, and cardiovascular death. | 1 year | 34 | 296 | NA | NA | NA | Cox regression | NA |
| 12 |  | Saeed Tofighi | 2023 | Iran | Single center | PCI | Including myocardial infarction, emerg-ent revascularization, hemodynamic instability, and all‐cause mortality. | 1 year | 610 | 4514 | 3160 | Random sampling | 1354 | Gradient Boosting Machine,  Random Forest,  Logistic Regression,  Deep Learning (DL) | NA |
| 13 |  | Long Tang | 2023 | China | Multicenter | PCI | The composite of cardiac mortality, re-currence of myocardial infarction, acu-te cardiac failure, stroke and stent thr-ombosis. | NA | 45 | 547 | 384 | Random sampling | 163 | Logistic regression | Age, Hypertension, Diabetes mellitus, Current smoking, Systolic blood pressure, Diastolic blood pressure, Hyperuricemia, Total cholesterol, NLR, PLR, Red blood cell, Platelet distribution width,  Gensini score. |
| 14 |  | Yu Tan | 2021 | China | Single center | PCI | Nonfatal MI or death. The secondary endpoint was death. | 6 months | 45 | 444 | NA | NA | NA | Cox regression | Age, Sex, Hypertension, Diabetes mellitus, eGFR, NT-proBNP, Log TMAO. |
| 15 |  | S. W. A. Sherazi | 2021 | South Korea | Multicenter | NA | Major adverse cardiovascular events (MACE) as cardiac death (CD), non-cardiac death (NCD), myocardial infarction (MI), re-percutaneous coronary intervention (re-PCI), and coronary artery bypass grafting (CABG). | 2 years | 713 | 11189 | 7832 | 5-fold stratified cross-validation | 3357 | Random forest, Extra tree, and gradient boosting | NA |
| 16 |  | Defeng Pan | 2021 | China | Single center | PCI | A compositeofcardiac death, recurrent AMI, unplanned revascularization,or rehospitalization for any cardiovascular disease, includingheart failure, nonfatal ischemic stroke, or unstable angina. | 1 year | 421 | 1958 | NA | bootstrap | NA | Logistic regression | Age, Diabetes mellitus, Low-density lipoprotein cholesterol, Uricacid, Lipoprotein (a), Left ventricular ejection fraction, Syntax score, Hypersensitive troponin T. |
| 17 |  | Quanmei Ma | 2020 | China | Single center | PCI | A composite of death, occurrence of new congestive heart failure, and myocardial reinfarction within one year. | 1 year | 17 | 157 | 109 | 10-fold cross-validation tuning | 48 | Logistic regression | cTnI, LVEF, IS, AAR, Remote myocardial T1 values. |
| 18 |  | Xiaobo Li | 2024 | China | Single center | NA | All-cause death, cardiogenic shock, target vessel re-revascularization, recurrent MI after treatment during the hospitalization, unstable angina pectoris, malignant arrhythmia, heart failure, stroke or transient ischemic attack (TIA), and stent throm­bosis events. | NA | 334 | 954 | 667 | Random sampling | 287 | Logistic regression | Age, Killip classification, BUN level, ECG-based  Diagnosis, left ventricular ejection fraction. |
| 19 |  | Joo Hee Jeong | 2024 | Korea | Multicenter | PCI, balloon angioplasty | A composite outcome requiring clinical intervention, including myocardial infarction, repeat percutaneous coronary intervention (PCI) of target or non-target vessel revascularization, coronary artery bypass graft, stent thrombosis, cerebrovascular accident (ischemic stroke or hemorrhagic stroke), and re-hospitalization due to heart failure aggravation. | 1 year | 568 | 9661 | 7246 | External validation | 2415 | Artificial neural network, Decision tree,  Logistic regression,  Naïve Bayes,  Random forest,  Support vector machine | NA |
| 20 |  | Xiaoxia Hou | 2021 | China | Multicenter | NA | Cardiovascular death, MI and ischaemic stroke occurring during the index hospitalisation. | NA | 888 | 15009 | 7329 | External validation | 7680 | Logistic regression | Age, White cell count, Killip class. |
| 21 |  | Jing Guo | 2023 | China | Single center |  | A composite of death, MI, and repeat revascularization. | 21.2 months | 20 | 210 |  |  |  | Logistic regression | TNF-α, IL-1β, IL-17A, History of diabetes mellitus, History of coronary heart disease, Symptom-to-balloon time. |
| 22 |  | Caoyang Fang | 2023 | China | Single center | PCI | The primary endpoint event as all-cause mortality. Secondary endpoints were reinfarction, emergency revascularization, cardiac arrest, acute heart failure, cardiogenic shock, malignant arrhythmia (including ventricular tachycardia/ventricular fibrillation, sinus arrest, high-grade or third-degree atrioventricular block), mechanical complications of myocardial infarction, stroke, and severe bleeding (hemoglobin decrease ≥3 g/L). | NA | 113 | 295 | 206 | Random sampling | 89 | Random forest | Serum YKL-40, albumin, blood glucose, hemoglobin, LVEF, and uric acid. |
| 23 |  | Z.-Y. Fan | 2021 | China | Single center | PCI | Heart failure, stroke, non-sustained ventricular tachycardia(VT), sustained VT,implantable cardioverteredefibrillator (ICD) implantation, and cardiac death. | NA | 32 | 155 | NA | NA | NA | Logistic regression | Perc.50%, S(4,-4)AngScMom, S(1,1)InvDfMom, S(0,2)DifEntrp, Horzl_LngREmph. |
| 24 |  | Emine Sebnem Durmaz | 2023 | Turkey | Single center | PCI | New-onset congestive heart failure, ventricular arrhythmia, and cardiac death. | NA | 28 | 60 | NA | Random sampling | NA | K-nearest neighbor,  AdaBoost,  Random forest,  Support vector machine,  Naive Bayes,  Gradient descent,  Neural network | Anterior MI, LVEDV, LVEDVi, LVESV, LVESVi, LVEF . |
| 25 |  | NAOMI NIARI DALIMUNTHE | 2022 | Indonesia | Single center | NA | Heart failure, cardiogenic shock, and all- cause mortality. | NA | 19 | 75 | NA | NA | NA | Logistic regression | NA |
| 26 |  | Yan Chen | 2024 | China | Single center | NA | All-cause death, myocardial reinfarction and heart failure hospitalization. | NA | 51 | 236 | 165 | 10-fold cross- validation | 71 | Cox regression | Kurtosis, Zone Variance, Idmn, Short Run Low Gray Level Emphasis Size Zone Non-Uniformity, Normalized MCC, Large Dependence Low Gray Level Emphasis, Low Gray Level Zone Emphasis, Small Dependence Low Gray Level Emphasis, Flatness, Small Dependence Low Gray Level Emphasis, Mean, Cluster Shade, Run Entropy, Small Area Low Gray Level Emphasis , Imc1, MCC, Cluster Prominence, Small Dependence Low Gray Level Emphasis , Low Gray Level Zone Emphasis. |
| 27 |  | Weiyao Chen | 2023 | China | Multicenter | PCI | MACEs, including all-cause mortality, non-fatal MI, and non-fatal ischemic stroke. | NA | 70 | 1247 | 1007 | External validation | 240 | Logistic regression | ST-segment deviation, Brain natriuretic peptide, Low-density lipoprotein cholesterol, Estimated glomerular filtration rate, Age, Hemoglobin, White blood cell count. |
| 28 |  | Adil Bayramoğlu | 2018 | Turkey | Multicenter | PCI | Stent thrombosis, target vessel revascularization. | 1 year | 217 | 1147 | 515 | External validation | 632 | Logistic regression | Age ≥65 years, Reperfusion time >4 h, High thrombus burden, Killip class ≥3, Long stent use |
